# Supplementary material for: The conservation value of human-modified landscapes for the world’s primates
Source: Nat Commun. 2019 Jan 11;10:152. doi: 10.1038/s41467-018-08139-0 (PMC6329842; doi:10.1038/s41467-018-08139-0)
Supplement: Supplementary file 1 — Supplementary Information [file 41467_2018_8139_MOESM1_ESM.pdf]

The conservation value of human-modified landscapes for the world's primates

Galán-Acedo et al.

Supplementary Information

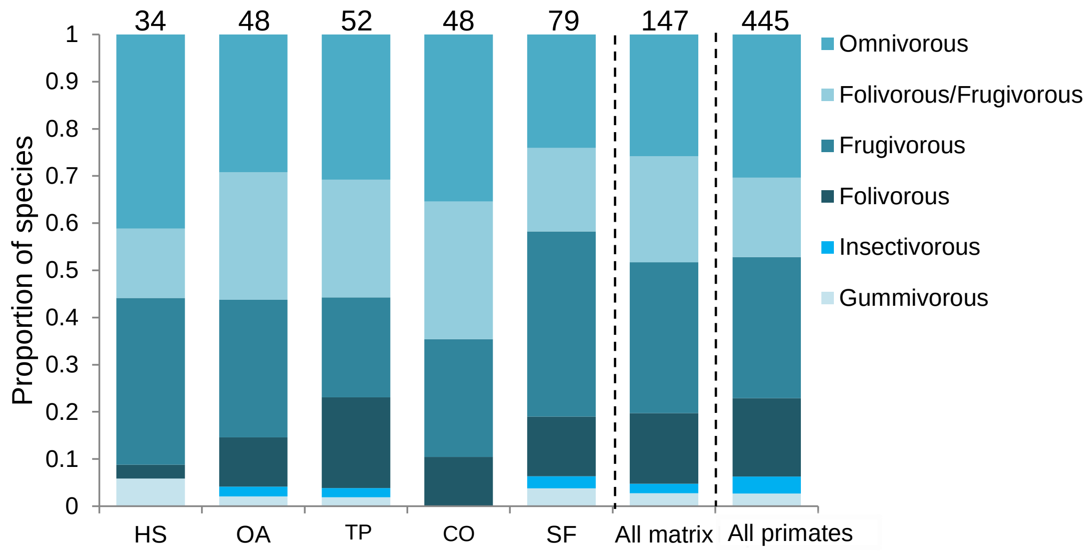

Supplementary Figure 1. The proportion (and total number above bars) of primate species within different dietary categories. We tested for differences in frequencies with Chi-square tests of goodness of fit (*$P \leq 0.05$, **$P \leq 0.01$) between primates using the matrix and all the world's primates, by separately assessing primates species that used different matrix types (HS = human settlements, OA = open areas, TP = tree plantations, CO = connectors, and SF = secondary forest). None of the tests were statistically significant. We excluded from the analyses those species for which there was no available information.

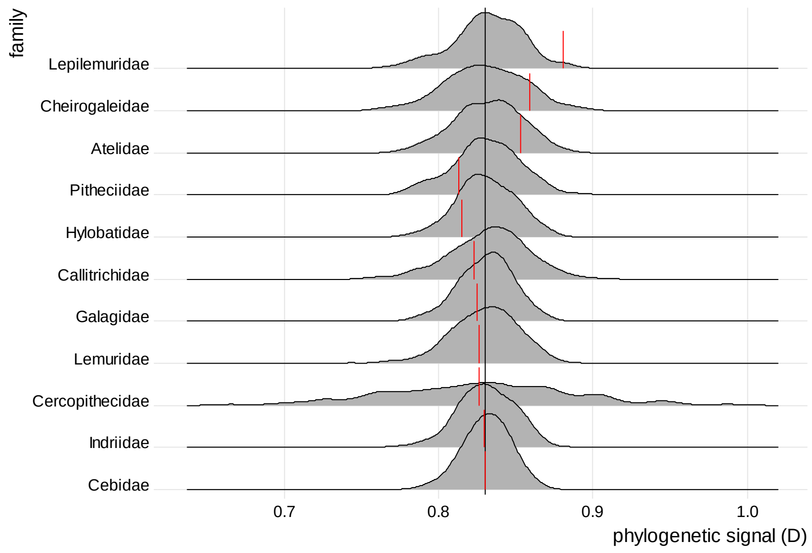

Supplementary Figure 2. Sensitivity analysis results for phylogenetic signal tests. Black line shows the estimate of D for the whole order. Red lines are the estimates after removing each family, and gray density plots show the distribution of D values obtained by randomly removing the number of species in the family 500 times.

Supplementary Note 1. Despite having a similar number of species in the tree, Lepilemuridae had, as most of the other families, the greatest influence on the parameter estimate when removing it from the analyses. We expected Cercopithecidae to have a large effect on parameter estimates because it includes a larger proportion of the species analyzed. However, the distribution of matrix use within this family is not distinguishable from the order as a whole. After correcting for clade size, only Lepilemuridae had a strong influence on our estimates of D. Removing this clade from the analysis led to a significant shift in phylogenetic signal towards a truly random pattern.

Supplementary Table 1. Primate species using the matrix. The sources of studies reporting the matrix use are included in the Reference column. Matrix: CO = connectors, HS = human settlements, OA = open areas, SF = secondary forests, TP = tree plantations; Realm: M_Africa = mainland Africa; activity: TR = travel, R = resting, F = foraging, NR = not reported; conservation status (IUCN): CR = critically endangered, EN = endangered, VU = vulnerable, NT = near threatened, LC = least concerned, DD = data deficient, NE = not evaluated; population trend (Pop_T): I = increasing, D = decreasing, S = stable, NA = no data available; diel activity (D_A): Di = diurnal, N = Nocturnal, Cath = cathemeral; locomotion: AR = arboreal locomotion, T = terrestrial locomotion; body mass (B_M): L = large (>10 kg), M = medium (2 – 10 kg), S = small (<2 kg); habitat (forest specialization): FS = forest specialist, N_FS = non forest specialist; trophic level (T_L), F_f = folivorous-frugivorous, Fol = folivorous, Fru = frugivorous, G = gummivorous, O = omnivorous, In = insectivorous. "N records" refers to the number of times that a given primate species was recorded using the matrix.

Matrix	Family[a]	*Species*	Realm	Activity	IUCN	Pop_T	D_A	Locomotion	B_M	Habitat	T_L	N records	Reference
HS	*Atelidae*	*Alouatta guariba*	Neotropics	TR	LC	D	DI	AR	M	FS	F_F	5	238
HS	*Callitrichidae*	*Callithrix jacchus*	Neotropics	F	LC	S	DI	AR	S	N_FS	G	2	243
HS	*Callitrichidae*	*Callithrix kuhlii*	Neotropics	F	NT	D	DI	AR	S	FS	O	2	210
HS	*Callitrichidae*	*Callithrix penicillata*	Neotropics	NR	LC	I	DI	AR	S	N_FS	G	7	237
HS	*Callitrichidae*	*Callithrix penicillata*	Neotropics	ALL	LC	I	DI	AR	S	N_FS	G	7	60
HS	*Callitrichidae*	*Callithrix penicillata*	Neotropics	F	LC	I	DI	AR	S	N_FS	G	7	243
HS	*Callitrichidae*	*Callithrix penicillata*	Neotropics	ALL	LC	I	DI	AR	S	N_FS	G	7	59
HS	*Callitrichidae*	*Callithrix penicillata*	Neotropics	ALL	LC	I	DI	AR	S	N_FS	G	7	61
HS	*Callitrichidae*	*Callithrix penicillata*	Neotropics	ALL	LC	I	DI	AR	S	N_FS	G	7	97
HS	*Cercopithecidae*	*Cercocebus torquatus*	M_Africa	NR	VU	D	DI	BOTH	M	FS	FRU	1	15
HS	*Cercopithecidae*	*Cercopithecus ascanius*	M_Africa	F	LC	NI	DI	AR	M	FS	FRU	12	94
HS	*Cercopithecidae*	*Cercopithecus erythrotis*	M_Africa	NR	VU	D	DI	AR	M	FS	FRU	1	15
HS	*Cercopithecidae*	*Cercopithecus mona*	M_Africa	NR	LC	NI	DI	AR	M	FS	FRU	2	15
HS	*Cercopithecidae*	*Cercopithecus nictitans*	M_Africa	NR	LC	D	DI	AR	M	FS	FRU	3	15
HS	*Cercopithecidae*	*Cercopithecus pogonias*	M_Africa	NR	LC	D	DI	AR	M	N_FS	FRU	3	15
HS	*Cercopithecidae*	*Cercopithecus sclateri*	M_Africa	F	VU	D	DI	AR	M	N_FS	FRU	1	12

HS	*Cercopithecidae*	*Chlorocebus aethiops*	M_Africa	F	LC	S	DI	T	M	N_FS	O	12	185
HS	*Cercopithecidae*	*Chlorocebus aethiops*	M_Africa	F	LC	S	DI	T	M	N_FS	O	12	220
HS	*Cercopithecidae*	*Chlorocebus pygerythrus*	M_Africa	NR	LC	S	DI	T	M	N_FS	O	1	154
HS	*Cercopithecidae*	*Colobus vellerosus*	M_Africa	NR	VU	NI	DI	AR	M	N_FS	FOL	1	236
HS	*Galagidae*	*Galago moholi*	M_Africa	ALL	LC	S	N	AR	S	N_FS	O	1	222
HS	*Callitrichidae*	*Leontocebus fuscicollis*	Neotropics	F	LC	D	DI	AR	S	FS	O	4	249
HS	*Cercopithecidae*	*Macaca cyclopis*	Asia	F	LC	S	DI	BOTH	M	FS	FRU	1	111
HS	*Cercopithecidae*	*Macaca fascicularis*	Asia	ALL	LC	D	DI	AR	M	FS	FRU	10	30
HS	*Cercopithecidae*	*Macaca fascicularis*	Asia	F	LC	D	DI	AR	M	FS	FRU	10	225
HS	*Cercopithecidae*	*Macaca fascicularis*	Asia	ALL	LC	D	DI	AR	M	FS	FRU	10	155
HS	*Cercopithecidae*	*Macaca fascicularis*	Asia	NR	LC	D	DI	AR	M	FS	FRU	10	82
HS	*Cercopithecidae*	*Macaca fuscata*	Asia	NR	LC	S	DI	BOTH	M	FS	F_F	6	113
HS	*Cercopithecidae*	*Macaca leonina*	Asia	ALL	VU	D	DI	AR	M	FS	FRU	1	3
HS	*Cercopithecidae*	*Macaca mulatta*	Asia	NR	LC	NI	DI	BOTH	M	N_FS	F_F	15	175
HS	*Cercopithecidae*	*Macaca mulatta*	Asia	ALL	LC	NI	DI	BOTH	M	N_FS	F_F	15	17
HS	*Cercopithecidae*	*Macaca mulatta*	Asia	ALL	LC	NI	DI	BOTH	M	N_FS	F_F	15	114
HS	*Cercopithecidae*	*Macaca mulatta*	Asia	ALL	LC	NI	DI	BOTH	M	N_FS	F_F	15	124
HS	*Cercopithecidae*	*Macaca mulatta*	Asia	F	LC	NI	DI	BOTH	M	N_FS	F_F	15	46
HS	*Cercopithecidae*	*Macaca mulatta*	Asia	NR	LC	NI	DI	BOTH	M	N_FS	F_F	15	213
HS	*Cercopithecidae*	*Macaca mulatta*	Asia	F	LC	NI	DI	BOTH	M	N_FS	F_F	15	45
HS	*Cercopithecidae*	*Macaca mulatta*	Asia	NR	LC	NI	DI	BOTH	M	N_FS	F_F	15	233
HS	*Cercopithecidae*	*Macaca mulatta*	Asia	NR	LC	NI	DI	BOTH	M	N_FS	F_F	15	231
HS	*Cercopithecidae*	*Macaca mulatta*	Asia	ALL	LC	NI	DI	BOTH	M	N_FS	F_F	15	85
HS	*Cercopithecidae*	*Macaca mulatta*	Asia	ALL	LC	NI	DI	BOTH	M	N_FS	F_F	15	147
HS	*Cercopithecidae*	*Macaca mulatta*	Asia	ALL	LC	NI	DI	BOTH	M	N_FS	F_F	15	232
HS	*Cercopithecidae*	*Macaca mulatta*	Asia	ALL	LC	NI	DI	BOTH	M	N_FS	F_F	15	195
HS	*Cercopithecidae*	*Macaca mulatta*	Asia	ALL	LC	NI	DI	BOTH	M	N_FS	F_F	15	125
HS	*Cercopithecidae*	*Macaca mulatta*	Asia	ALL	LC	NI	DI	BOTH	M	N_FS	F_F	15	102
HS	*Cercopithecidae*	*Macaca radiata*	Asia	ALL	LC	D	DI	BOTH	M	N_FS	FRU	11	48
HS	*Cercopithecidae*	*Macaca radiata*	Asia	ALL	LC	D	DI	BOTH	M	N_FS	FRU	11	49
HS	*Cercopithecidae*	*Macaca radiata*	Asia	ALL	LC	D	DI	BOTH	M	N_FS	FRU	11	47

HS	*Cercopithecidae*	*Macaca radiata*	Asia	ALL	LC	D	DI	BOTH	M	N_FS	FRU	11	202
HS	*Cercopithecidae*	*Macaca radiata*	Asia	ALL	LC	D	DI	BOTH	M	N_FS	FRU	11	228
HS	*Cercopithecidae*	*Macaca sínica*	Asia	TR	EN	D	DI	AR	M	FS	FRU	1	63
HS	*Cercopithecidae*	*Macaca sylvanus*	M_Africa	F	EN	D	DI	BOTH	L	N_FS	O	2	141
HS	*Cercopithecidae*	*Macaca sylvanus*	M_Africa	NR	EN	D	DI	BOTH	L	N_FS	O	2	82
HS	*Cercopithecidae*	*Mandrillus leucophaeus*	M_Africa	NR	EN	NI	DI	BOTH	L	N_FS	O	1	15
HS	*Lorisidae*	*Nycticebus coucang*	Asia	R	VU	D	N	AR	S	FS	O	2	70
HS	*Hominidae*	*Pan troglodytes*	M_Africa	NR	EN	D	DI	BOTH	L	N_FS	O	17	15
HS	*Hominidae*	*Pan troglodytes*	M_Africa	F	EN	D	DI	BOTH	L	N_FS	O	17	62
HS	*Cercopithecidae*	*Papio anubis*	M_Africa	F	LC	I	DI	T	L	N_FS	O	11	217
HS	*Cercopithecidae*	*Papio cynocephalus*	M_Africa	F	LC	S	DI	T	L	N_FS	O	3	4
HS	*Cercopithecidae*	*Papio ursinus*	M_Africa	ALL	LC	S	DI	T	L	N_FS	O	7	110
HS	*Cercopithecidae*	*Papio ursinus*	M_Africa	F	LC	S	DI	T	L	N_FS	O	7	75
HS	*Cercopithecidae*	*Papio ursinus*	M_Africa	F	LC	S	DI	T	L	N_FS	O	7	109
HS	*Cercopithecidae*	*Papio ursinus*	M_Africa	F	LC	S	DI	T	L	N_FS	O	7	117
HS	*Cercopithecidae*	*Papio ursinus*	M_Africa	ALL	LC	S	DI	T	L	N_FS	O	7	108
HS	*Cercopithecidae*	*Piliocolobus pennantii*	M_Africa	NR	EN	D	DI	AR	M	FS	F_F	1	15
HS	*Callitrichidae*	*Saguinus mystax*	Neotropics	F	LC	S	DI	AR	S	FS	O	4	249
HS	*Cebidae*	*Sapajus libidinosus*	Neotropics	ALL	LC	D	DI	AR	M	N_FS	O	3	89
HS	*Cercopithecidae*	*Semnopithecus entellus*	Asia	NR	LC	D	DI	T	L	N_FS	F_F	8	175
HS	*Cercopithecidae*	*Semnopithecus entellus*	Asia	ALL	LC	D	DI	T	L	N_FS	F_F	8	247
HS	*Cercopithecidae*	*Semnopithecus entellus*	Asia	NR	LC	D	DI	T	L	N_FS	F_F	8	1
HS	*Cercopithecidae*	*Semnopithecus entellus*	Asia	NR	LC	D	DI	T	L	N_FS	F_F	8	213
HS	*Cercopithecidae*	*Semnopithecus entellus*	Asia	ALL	LC	D	DI	T	L	N_FS	F_F	8	180
HS	*Cercopithecidae*	*Semnopithecus entellus*	Asia	ALL	LC	D	DI	T	L	N_FS	F_F	8	147
HS	*Cercopithecidae*	*Semnopithecus entellus*	Asia	ALL	LC	D	DI	T	L	N_FS	F_F	8	162
OA	*Atelidae*	*Alouatta caraya*	Neotropics	TR	LC	D	DI	AR	M	N_FS	F_F	2	22
OA	*Atelidae*	*Alouatta palliata*	Neotropics	F	LC	NI	DI	AR	M	FS	F_F	25	229
OA	*Atelidae*	*Alouatta palliata*	Neotropics	TR	LC	NI	DI	AR	M	FS	F_F	25	95
OA	*Atelidae*	*Alouatta pigra*	Neotropics	R	EN	D	DI	AR	M	FS	F_F	12	198
OA	*Atelidae*	*Alouatta pigra*	Neotropics	F	EN	D	DI	AR	M	FS	F_F	12	196

OA	*Atelidae*	*Alouatta pigra*	Neotropics	TR	EN	D	DI	AR	M	FS	F_F	12	18
OA	*Atelidae*	*Alouatta seniculus*	Neotropics	TR	LC	NI	DI	AR	M	N_FS	F_F	7	34
OA	*Atelidae*	*Brachyteles arachnoides*	Neotropics	TR	EN	D	DI	AR	M	FS	F_F	1	132
OA	*Atelidae*	*Brachyteles hypoxanthus*	Neotropics	TR	CR	D	DI	AR	M	FS	F_F	1	57
OA	*Cebidae*	*Cebus capucinus*	Neotropics	F	LC	NI	DI	AR	M	FS	O	15	151
OA	*Cebidae*	*Cebus capucinus*	Neotropics	F	LC	NI	DI	AR	M	FS	O	15	229
OA	*Cebidae*	*Cebus capucinus*	Neotropics	TR	LC	NI	DI	AR	M	FS	O	15	72
OA	*Cebidae*	*Cebus capucinus*	Neotropics	TR	LC	NI	DI	AR	M	FS	O	15	41
OA	*Cebidae*	*Cebus capucinus*	Neotropics	F	LC	NI	DI	AR	M	FS	O	15	96
OA	*Cercopithecidae*	*Cercocebus galeritus*	M_Africa	F	EN	D	DI	BOTH	M	FS	FRU	3	164
OA	*Cercopithecidae*	*Cercopithecus ascanius*	M_Africa	F	LC	NI	DI	AR	M	FS	FRU	12	248
OA	*Cercopithecidae*	*Cercopithecus ascanius*	M_Africa	F	LC	NI	DI	AR	M	FS	FRU	12	192
OA	*Cercopithecidae*	*Cercopithecus ascanius*	M_Africa	F	LC	NI	DI	AR	M	FS	FRU	12	168
OA	*Cercopithecidae*	*Cercopithecus ascanius*	M_Africa	F	LC	NI	DI	AR	M	FS	FRU	12	83
OA	*Cercopithecidae*	*Cercopithecus campbelli*	M_Africa	F	LC	NI	DI	AR	M	N_FS	FRU	5	250
OA	*Cercopithecidae*	*Cercopithecus cephus*	M_Africa	NR	LC	NI	DI	AR	M	FS	FRU	2	146
OA	*Cercopithecidae*	*Cercopithecus mitis*	M_Africa	F	LC	D	DI	AR	M	FS	O	10	248
OA	*Cercopithecidae*	*Cercopithecus mitis*	M_Africa	F	LC	D	DI	AR	M	FS	O	10	28
OA	*Cercopithecidae*	*Cercopithecus mitis*	M_Africa	F	LC	D	DI	AR	M	FS	O	10	164
OA	*Cercopithecidae*	*Cercopithecus mitis*	M_Africa	F	LC	D	DI	AR	M	FS	O	10	178
OA	*Cercopithecidae*	*Cercopithecus mona*	M_Africa	NR	LC	NI	DI	AR	M	FS	FRU	2	146
OA	*Cercopithecidae*	*Cercopithecus nictitans*	M_Africa	NR	LC	D	DI	AR	M	FS	FRU	3	146
OA	*Cercopithecidae*	*Chlorocebus aethiops*	M_Africa	F	LC	S	DI	T	M	N_FS	O	12	248
OA	*Cercopithecidae*	*Chlorocebus aethiops*	M_Africa	F	LC	S	DI	T	M	N_FS	O	12	25
OA	*Cercopithecidae*	*Chlorocebus aethiops*	M_Africa	F	LC	S	DI	T	M	N_FS	O	12	220
OA	*Cercopithecidae*	*Chlorocebus aethiops*	M_Africa	F	LC	S	DI	T	M	N_FS	O	12	164
OA	*Cercopithecidae*	*Chlorocebus aethiops*	M_Africa	F	LC	S	DI	T	M	N_FS	O	12	120
OA	*Cercopithecidae*	*Chlorocebus djamdjamensis*	M_Africa	F	VU	D	DI	BOTH	M	FS	FOL	1	156
OA	*Cercopithecidae*	*Colobus angolensis*	M_Africa	TR	LC	NI	DI	AR	M	FS	FOL	5	6
OA	*Lemuridae*	*Lemur catta*	Madagascar	F	EN	D	DI	BOTH	M	N_FS	F_F	1	128
OA	*Cercopithecidae*	*Lophocebus ugandae*	M_Africa	F	NE	NI	DI	AR	M	FS	F_F	2	83

OA	*Cercopithecidae*	*Macaca assamensis*	Asia	F	NT	D	DI	AR	M	FS	FRU	1	207
OA	*Cercopithecidae*	*Macaca fascicularis*	Asia	F	LC	D	DI	AR	M	FS	FRU	10	143
OA	*Cercopithecidae*	*Macaca fuscata*	Asia	R	LC	S	DI	BOTH	M	FS	F_F	6	64
OA	*Cercopithecidae*	*Macaca fuscata*	Asia	F	LC	S	DI	BOTH	M	FS	F_F	6	256
OA	*Cercopithecidae*	*Macaca fuscata*	Asia	ALL	LC	S	DI	BOTH	M	FS	F_F	6	112
OA	*Cercopithecidae*	*Macaca nemestrina*	Asia	F	VU	D	DI	T	M	FS	FRU	4	143
OA	*Cercopithecidae*	*Macaca ochreata*	Asia	F	VU	D	DI	BOTH	M	FS	FRU	3	199
OA	*Cercopithecidae*	*Macaca ochreata*	Asia	F	VU	D	DI	BOTH	M	FS	FRU	3	200
OA	*Cercopithecidae*	*Macaca radiata*	Asia	F	LC	D	DI	BOTH	M	N_FS	FRU	11	226
OA	*Cercopithecidae*	*Macaca radiata*	Asia	F	LC	D	DI	BOTH	M	N_FS	FRU	11	227
OA	*Cercopithecidae*	*Macaca tonkeana*	Asia	F	VU	D	DI	BOTH	L	FS	FRU	1	208
OA	*Cercopithecidae*	*Miopithecus talapoin*	M_Africa	NR	LC	NI	DI	BOTH	S	FS	O	2	146
OA	*Lorisidae*	*Nycticebus javanicus*	Asia	NR	CR	D	N	AR	S	FS	G	2	245
OA	*Hominidae*	*Pan troglodytes*	M_Africa	F	EN	D	DI	BOTH	L	N_FS	O	17	36
OA	*Hominidae*	*Pan troglodytes*	M_Africa	F	EN	D	DI	BOTH	L	N_FS	O	17	107
OA	*Hominidae*	*Pan troglodytes*	M_Africa	F	EN	D	DI	BOTH	L	N_FS	O	17	192
OA	*Hominidae*	*Pan troglodytes*	M_Africa	F	EN	D	DI	BOTH	L	N_FS	O	17	168
OA	*Hominidae*	*Pan troglodytes*	M_Africa	ALL	EN	D	DI	BOTH	L	N_FS	O	17	241
OA	*Hominidae*	*Pan troglodytes*	M_Africa	F	EN	D	DI	BOTH	L	N_FS	O	17	20
OA	*Hominidae*	*Pan troglodytes*	M_Africa	F	EN	D	DI	BOTH	L	N_FS	O	17	105
OA	*Cercopithecidae*	*Papio anubis*	M_Africa	F	LC	I	DI	T	L	N_FS	O	11	131
OA	*Cercopithecidae*	*Papio anubis*	M_Africa	F	LC	I	DI	T	L	N_FS	O	11	248
OA	*Cercopithecidae*	*Papio anubis*	M_Africa	ALL	LC	I	DI	T	L	N_FS	O	11	241
OA	*Cercopithecidae*	*Papio anubis*	M_Africa	NR	LC	I	DI	T	L	N_FS	O	11	218
OA	*Cercopithecidae*	*Papio anubis*	M_Africa	F	LC	I	DI	T	L	N_FS	O	11	164
OA	*Cercopithecidae*	*Papio anubis*	M_Africa	F	LC	I	DI	T	L	N_FS	O	11	105
OA	*Cercopithecidae*	*Papio cynocephalus*	M_Africa	F	LC	S	DI	T	L	N_FS	O	3	192
OA	*Cercopithecidae*	*Papio cynocephalus*	M_Africa	F	LC	S	DI	T	L	N_FS	O	3	168
OA	*Cercopithecidae*	*Papio hamadryas*	M_Africa	F	LC	I	DI	T	L	N_FS	O	4	106
OA	*Cercopithecidae*	*Papio hamadryas*	M_Africa	TR	LC	I	DI	T	L	N_FS	O	4	187
OA	*Cercopithecidae*	*Papio ursinus*	M_Africa	ALL	LC	S	DI	T	L	N_FS	O	7	108

OA	*Cercopithecidae*	*Piliocolobus badius*	M_Africa	TR	EN	D	DI	AR	M	FS	F_F	5	93
OA	*Pitheciidae*	*Plecturocebus modestus*	Neotropics	TR	EN	D	DI	AR	S	N_FS	FRU	1	76
OA	*Pitheciidae*	*Plecturocebus Moloch*	Neotropics	TR	LC	NI	DI	AR	S	FS	O	2	77
OA	*Pitheciidae*	*Plecturocebus olallae*	Neotropics	TR	EN	D	DI	AR	S	N_FS	F_F	2	76
OA	*Cercopithecidae*	*Presbytis thomasi*	Asia	F	VU	D	DI	AR	M	FS	F_F	2	143
OA	*Indriidae*	*Propithecus verreauxi*	Madagascar	F	EN	D	DI	AR	M	N_FS	F_F	4	86
OA	*Cercopithecidae*	*Rungwecebus kipunji*	M_Africa	F	CR	D	DI	AR	L	FS	O	2	28
OA	*Callitrichidae*	*Saguinus leucopus*	Neotropics	TR	EN	D	DI	AR	S	FS	FRU	7	53
OA	*Callitrichidae*	*Saguinus leucopus*	Neotropics	TR	EN	D	DI	AR	S	FS	FRU	7	127
OA	*Cebidae*	*Saimiri sciureus*	Neotropics	TR	LC	D	DI	AR	S	FS	O	3	34
OA	*Cebidae*	*Sapajus apella*	Neotropics	F	LC	D	DI	AR	M	N_FS	O	11	190
OA	*Cebidae*	*Sapajus apella*	Neotropics	TR	LC	D	DI	AR	M	N_FS	O	11	34
OA	*Cebidae*	*Sapajus libidinosus*	Neotropics	F	LC	D	DI	AR	M	N_FS	O	3	81
OA	*Tarsiidae*	*Tarsius dentatus*	Asia	TR	VU	D	N	AR	S	FS	IN	3	158
OA	*Cercopithecidae*	*Thercopithecus gelada*	M_Africa	TR	LC	D	DI	T	L	N_FS	FOL	1	121
OA	*Cercopithecidae*	*Trachypithecus germaini*	Asia	F	EN	D	DI	AR	M	FS	FOL	2	143
OA	*Cercopithecidae*	*Trachypithecus pileatus*	Asia	NR	VU	D	DI	AR	L	FS	FOL	1	219
TP	*Atelidae*	*Alouatta palliata*	Neotropics	ALL	LC	NI	DI	AR	M	FS	F_F	25	253
TP	*Atelidae*	*Alouatta palliata*	Neotropics	ALL	LC	NI	DI	AR	M	FS	F_F	25	165
TP	*Atelidae*	*Alouatta palliata*	Neotropics	ALL	LC	NI	DI	AR	M	FS	F_F	25	149
TP	*Atelidae*	*Alouatta palliata*	Neotropics	ALL	LC	NI	DI	AR	M	FS	F_F	25	68
TP	*Atelidae*	*Alouatta palliata*	Neotropics	NR	LC	NI	DI	AR	M	FS	F_F	25	69
TP	*Atelidae*	*Alouatta palliata*	Neotropics	F	LC	NI	DI	AR	M	FS	F_F	25	69
TP	*Atelidae*	*Alouatta pigra*	Neotropics	F	EN	D	DI	AR	M	FS	F_F	12	258
TP	*Atelidae*	*Alouatta pigra*	Neotropics	ALL	EN	D	DI	AR	M	FS	F_F	12	198
TP	*Atelidae*	*Alouatta pigra*	Neotropics	ALL	EN	D	DI	AR	M	FS	F_F	12	197
TP	*Atelidae*	*Alouatta pigra*	Neotropics	NR	EN	D	DI	AR	M	FS	F_F	12	69
TP	*Aotidae*	*Aotus lemurinus*	Neotropics	F	VU	D	N	AR	S	FS	O	2	100
TP	*Atelidae*	*Ateles geoffroyi*	Neotropics	NR	EN	D	DI	AR	M	FS	FRU	19	68
TP	*Atelidae*	*Ateles geoffroyi*	Neotropics	ALL	EN	D	DI	AR	M	FS	FRU	19	69
TP	*Indriidae*	*Avahi laniger*	Madagascar	F	VU	D	N	AR	S	FS	FOL	2	86

TP	*Callitrichidae*	*Callithrix kuhlii*	Neotropics	ALL	NT	D	DI	AR	S	FS	O	2	240
TP	*Cebidae*	*Cebus capucinus*	Neotropics	F	LC	NI	DI	AR	M	FS	O	15	151
TP	*Cebidae*	*Cebus capucinus*	Neotropics	NR	LC	NI	DI	AR	M	FS	O	15	69
TP	*Cercopithecidae*	*Cercocebus galeritus*	M_Africa	F	EN	D	DI	BOTH	M	FS	FRU	3	164
TP	*Cercopithecidae*	*Cercopithecus ascanius*	M_Africa	F	LC	NI	DI	AR	M	FS	FRU	12	13
TP	*Cercopithecidae*	*Cercopithecus ascanius*	M_Africa	F	LC	NI	DI	AR	M	FS	FRU	12	83
TP	*Cercopithecidae*	*Cercopithecus campbelli*	M_Africa	F	LC	NI	DI	AR	M	N_FS	FRU	5	250
TP	*Cercopithecidae*	*Cercopithecus mitis*	M_Africa	F	LC	D	DI	AR	M	FS	O	10	164
TP	*Cercopithecidae*	*Cercopithecus mitis*	M_Africa	F	LC	D	DI	AR	M	FS	O	10	178
TP	*Cheirogaleidae*	*Cheirogaleus major*	Madagascar	ALL	DD	D	N	AR	S	FS	F_F	2	86
TP	*Cercopithecidae*	*Chlorocebus aethiops*	M_Africa	F	LC	S	DI	T	M	N_FS	O	12	25
TP	*Cercopithecidae*	*Chlorocebus aethiops*	M_Africa	F	LC	S	DI	T	M	N_FS	O	12	220
TP	*Cercopithecidae*	*Chlorocebus aethiops*	M_Africa	F	LC	S	DI	T	M	N_FS	O	12	164
TP	*Cercopithecidae*	*Chlorocebus aethiops*	M_Africa	F	LC	S	DI	T	M	N_FS	O	12	120
TP	*Cercopithecidae*	*Colobus angolensis*	M_Africa	ALL	LC	NI	DI	AR	M	FS	FOL	5	6
TP	*Cercopithecidae*	*Colobus angolensis*	M_Africa	F	LC	NI	DI	AR	M	FS	FOL	5	7
TP	*Lemuridae*	*Eulemur fulvus*	Madagascar	ALL	NT	D	CATH	AR	M	FS	F_F	2	86
TP	*Lemuridae*	*Hapalemur griseus*	Madagascar	F	VU	D	DI	AR	S	FS	FOL	2	86
TP	*Hylobatidae*	*Hylobates lar*	Asia	NR	EN	D	DI	AR	M	FS	FRU	2	11
TP	*Indriidae*	*Indri indri*	Madagascar	TR	CR	D	DI	AR	M	FS	FOL	1	86
TP	*Callitrichidae*	*Leontopithecus chrysomelas*	Neotropics	ALL	EN	D	DI	AR	S	FS	O	4	176
TP	*Callitrichidae*	*Leontopithecus chrysomelas*	Neotropics	ALL	EN	D	DI	AR	S	FS	O	4	177
TP	*Callitrichidae*	*Leontopithecus chrysomelas*	Neotropics	ALL	EN	D	DI	AR	S	FS	O	4	240
TP	*Lepilemuridae*	*Lepilemur mustelinus*	Madagascar	ALL	NT	D	N	AR	S	FS	FOL	1	86
TP	*Cercopithecidae*	*Lophocebus ugandae*	M_Africa	F	NE	NI	DI	AR	M	FS	F_F	2	83
TP	*Cercopithecidae*	*Macaca fascicularis*	Asia	NR	LC	D	DI	AR	M	FS	FRU	10	167
TP	*Cercopithecidae*	*Macaca fascicularis*	Asia	NR	LC	D	DI	AR	M	FS	FRU	10	11
TP	*Cercopithecidae*	*Macaca fascicularis*	Asia	F	LC	D	DI	AR	M	FS	FRU	10	143
TP	*Cercopithecidae*	*Macaca fuscata*	Asia	F	LC	S	DI	BOTH	M	FS	F_F	6	256
TP	*Cercopithecidae*	*Macaca fuscata*	Asia	F	LC	S	DI	BOTH	M	FS	F_F	6	221
TP	*Cercopithecidae*	*Macaca nemestrina*	Asia	NR	VU	D	DI	T	M	FS	FRU	4	11

TP	*Cercopithecidae*	*Macaca nemestrina*	Asia	F	VU	D	DI	T	M	FS	FRU	4	143
TP	*Cercopithecidae*	*Macaca ochreata*	Asia	F	VU	D	DI	BOTH	M	FS	FRU	3	200
TP	*Cercopithecidae*	*Macaca radiata*	Asia	NR	LC	D	DI	BOTH	M	N_FS	FRU	11	119
TP	*Cercopithecidae*	*Macaca radiata*	Asia	F	LC	D	DI	BOTH	M	N_FS	FRU	11	226
TP	*Cercopithecidae*	*Macaca radiata*	Asia	F	LC	D	DI	BOTH	M	N_FS	FRU	11	227
TP	*Cercopithecidae*	*Macaca radiata*	Asia	F	LC	D	DI	BOTH	M	N_FS	FRU	11	21
TP	*Cheirogaleidae*	*Microcebus murinus*	Madagascar	NR	LC	D	N	AR	S	N_FS	O	3	8
TP	*Cheirogaleidae*	*Microcebus rufus*	Madagascar	R	VU	D	N	AR	S	FS	O	1	86
TP	*Cercopithecidae*	*Nasalis larvatus*	Asia	TR	EN	D	DI	AR	L	FS	F_F	1	23
TP	*Lorisidae*	*Nycticebus coucang*	Asia	NR	VU	D	N	AR	S	FS	O	2	11
TP	*Lorisidae*	*Nycticebus javanicus*	Asia	NR	CR	D	N	AR	S	FS	G	2	245
TP	*Hominidae*	*Pan troglodytes*	M_Africa	ALL	EN	D	DI	BOTH	L	N_FS	O	17	241
TP	*Hominidae*	*Pan troglodytes*	M_Africa	F	EN	D	DI	BOTH	L	N_FS	O	17	20
TP	*Hominidae*	*Pan troglodytes*	M_Africa	F	EN	D	DI	BOTH	L	N_FS	O	17	37
TP	*Hominidae*	*Pan troglodytes*	M_Africa	F	EN	D	DI	BOTH	L	N_FS	O	17	101
TP	*Cercopithecidae*	*Papio anubis*	M_Africa	ALL	LC	I	DI	T	L	N_FS	O	11	241
TP	*Cercopithecidae*	*Papio anubis*	M_Africa	F	LC	I	DI	T	L	N_FS	O	11	164
TP	*Cercopithecidae*	*Papio hamadryas*	M_Africa	F	LC	I	DI	T	L	N_FS	O	4	104
TP	*Cercopithecidae*	*Papio ursinus*	M_Africa	ALL	LC	S	DI	T	L	N_FS	O	7	108
TP	*Cercopithecidae*	*Piliocolobus gordonorum*	M_Africa	ALL	EN	D	DI	AR	M	FS	F_F	1	173
TP	*Cercopithecidae*	*Piliocolobus rufomitratus*	M_Africa	F	EN	D	DI	AR	M	FS	FOL	1	164
TP	*Hominidae*	*Pongo abelii*	Asia	ALL	CR	D	DI	AR	L	FS	FRU	2	31
TP	*Hominidae*	*Pongo abelii*	Asia	F	CR	D	DI	AR	L	FS	FRU	2	143
TP	*Hominidae*	*Pongo pygmaeus*	Asia	F	EN	D	DI	AR	L	FS	FRU	2	206
TP	*Cercopithecidae*	*Presbytis thomasi*	Asia	F	VU	D	DI	AR	M	FS	F_F	2	143
TP	*Indriidae*	*Propithecus verreauxi*	Madagascar	ALL	EN	D	DI	AR	M	N_FS	F_F	4	86
TP	*Indriidae*	*Propithecus verreauxi*	Madagascar	F	EN	D	DI	AR	M	N_FS	F_F	4	86
TP	*Cebidae*	*Saimiri oerstedii*	Neotropics	NR	VU	D	DI	AR	S	FS	O	3	69
TP	*Cebidae*	*Sapajus nigritus*	Neotropics	F	NT	D	DI	AR	M	FS	O	4	136
TP	*Cebidae*	*Sapajus nigritus*	Neotropics	F	NT	D	DI	AR	M	FS	O	4	160
TP	*Cebidae*	*Sapajus nigritus*	Neotropics	F	NT	D	DI	AR	M	FS	O	4	161

TP	*Cebidae*	*Sapajus xanthosternos*	Neotropics	F	CR	D	DI	AR	M	FS	O	4	32
TP	*Cercopithecidae*	*Semnopithecus entellus*	Asia	NR	LC	D	DI	T	L	N_FS	F_F	8	119
TP	*Cercopithecidae*	*Semnopithecus vetulus*	Asia	F	EN	D	DI	AR	M	FS	F_F	10	182
TP	*Cercopithecidae*	*Semnopithecus vetulus*	Asia	NR	EN	D	DI	AR	M	FS	F_F	10	211
TP	*Cercopithecidae*	*Semnopithecus vetulus*	Asia	F	EN	D	DI	AR	M	FS	F_F	10	55
TP	*Cercopithecidae*	*Semnopithecus vetulus*	Asia	F	EN	D	DI	AR	M	FS	F_F	10	56
TP	*Cercopithecidae*	*Semnopithecus vetulus*	Asia	F	EN	D	DI	AR	M	FS	F_F	10	215
TP	*Cercopithecidae*	*Semnopithecus vetulus*	Asia	F	EN	D	DI	AR	M	FS	F_F	10	67
TP	*Hylobatidae*	*Symphalangus syndactylus*	Asia	NR	EN	D	DI	AR	L	FS	F_F	1	11
TP	*Tarsiidae*	*Tarsius dentatus*	Asia	TR	VU	D	N	AR	S	FS	IN	3	158
TP	*Tarsiidae*	*Tarsius dentatus*	Asia	R	VU	D	N	AR	S	FS	IN	3	159
TP	*Cercopithecidae*	*Trachypithecus auratus*	Asia	TR	VU	D	DI	AR	M	FS	FOL	2	170
TP	*Cercopithecidae*	*Trachypithecus cristatus*	Asia	NR	NT	D	DI	AR	M	FS	FOL	1	11
TP	*Cercopithecidae*	*Trachypithecus germaini*	Asia	F	EN	D	DI	AR	M	FS	FOL	2	143
TP	*Cercopithecidae*	*Trachypithecus obscurus*	Asia	NR	NT	D	DI	AR	M	FS	FOL	2	11
CO	*Atelidae*	*Alouatta caraya*	Neotropics	ALL	LC	D	DI	AR	M	N_FS	F_F	2	2
CO	*Atelidae*	*Alouatta guariba*	Neotropics	TR	LC	D	DI	AR	M	FS	F_F	5	238
CO	*Atelidae*	*Alouatta guariba*	Neotropics	TR	LC	D	DI	AR	M	FS	F_F	5	138
CO	*Atelidae*	*Alouatta palliata*	Neotropics	ALL	LC	NI	DI	AR	M	FS	F_F	25	10
CO	*Atelidae*	*Alouatta palliata*	Neotropics	NR	LC	NI	DI	AR	M	FS	F_F	25	150
CO	*Atelidae*	*Alouatta palliata*	Neotropics	NR	LC	NI	DI	AR	M	FS	F_F	25	69
CO	*Atelidae*	*Alouatta palliata*	Neotropics	NR	LC	NI	DI	AR	M	FS	F_F	25	69
CO	*Atelidae*	*Alouatta palliata*	Neotropics	TR	LC	NI	DI	AR	M	FS	F_F	21	152
CO	*Atelidae*	*Alouatta palliata*	Neotropics	ALL	LC	NI	DI	AR	M	FS	F_F	25	234
CO	*Atelidae*	*Alouatta palliata*	Neotropics	TR	LC	NI	DI	AR	M	FS	F_F	25	73
CO	*Atelidae*	*Alouatta palliata*	Neotropics	TR	LC	NI	DI	AR	M	FS	F_F	25	58
CO	*Atelidae*	*Alouatta palliata*	Neotropics	TR	LC	NI	DI	AR	M	FS	F_F	25	137
CO	*Atelidae*	*Alouatta palliata*	Neotropics	TR	LC	NI	DI	AR	M	FS	F_F	25	166
CO	*Atelidae*	*Alouatta palliata*	Neotropics	ALL	LC	NI	DI	AR	M	FS	F_F	25	139
CO	*Atelidae*	*Alouatta pigra*	Neotropics	ALL	EN	D	DI	AR	M	FS	F_F	12	197
CO	*Atelidae*	*Alouatta pigra*	Neotropics	ALL	EN	D	DI	AR	M	FS	F_F	12	198

CO	*Atelidae*	*Alouatta pigra*	Neotropics	NR	EN	D	DI	AR	M	FS	F_F	12	69
CO	*Atelidae*	*Alouatta pigra*	Neotropics	F	EN	D	DI	AR	M	FS	F_F	12	9
CO	*Atelidae*	*Alouatta seniculus*	Neotropics	F	LC	NI	DI	AR	M	N_FS	F_F	7	194
CO	*Atelidae*	*Alouatta seniculus*	Neotropics	F	LC	NI	DI	AR	M	N_FS	F_F	7	34
CO	*Atelidae*	*Alouatta seniculus*	Neotropics	ALL	LC	NI	DI	AR	M	N_FS	F_F	7	169
CO	*Atelidae*	*Alouatta seniculus*	Neotropics	TR	LC	NI	DI	AR	M	N_FS	F_F	7	29
CO	*Aotidae*	*Aotus lemurinus*	Neotropics	F	VU	D	N	AR	S	FS	O	2	38
CO	*Atelidae*	*Ateles belzebuth*	Neotropics	TR	EN	D	DI	AR	M	FS	FRU	2	98
CO	*Atelidae*	*Ateles geoffroyi*	Neotropics	NR	EN	D	DI	AR	M	FS	FRU	19	157
CO	*Atelidae*	*Ateles geoffroyi*	Neotropics	NR	EN	D	DI	AR	M	FS	FRU	19	69
CO	*Atelidae*	*Ateles geoffroyi*	Neotropics	F	EN	D	DI	AR	M	FS	FRU	19	191
CO	*Atelidae*	*Ateles geoffroyi*	Neotropics	F	EN	D	DI	AR	M	FS	FRU	19	191
CO	*Atelidae*	*Ateles geoffroyi*	Neotropics	F	EN	D	DI	AR	M	FS	FRU	19	191
CO	*Atelidae*	*Ateles geoffroyi*	Neotropics	NR	EN	D	DI	AR	M	FS	FRU	19	254
CO	*Atelidae*	*Ateles geoffroyi*	Neotropics	TR	EN	D	DI	AR	M	FS	FRU	19	58
CO	*Atelidae*	*Ateles geoffroyi*	Neotropics	TR	EN	D	DI	AR	M	FS	FRU	19	166
CO	*Atelidae*	*Ateles geoffroyi*	Neotropics	TR	EN	D	DI	AR	M	FS	FRU	19	139
CO	*Indriidae*	*Avahi laniger*	Madagascar	TR	VU	D	N	AR	S	FS	FOL	2	145
CO	*Cebidae*	*Cebus capucinus*	Neotropics	NR	LC	NI	DI	AR	M	FS	O	15	69
CO	*Cebidae*	*Cebus capucinus*	Neotropics	F	LC	NI	DI	AR	M	FS	O	15	252
CO	*Cebidae*	*Cebus capucinus*	Neotropics	TR	LC	NI	DI	AR	M	FS	O	15	142
CO	*Atelidae*	*Cebus capucinus*	Neotropics	TR	LC	NI	DI	AR	M	FS	O	15	139
CO	*Cercopithecidae*	*Cercopithecus ascanius*	M_Africa	TR	LC	NI	DI	AR	M	FS	FRU	12	44
CO	*Cercopithecidae*	*Cercopithecus ascanius*	M_Africa	TR	LC	NI	DI	AR	M	FS	FRU	12	179
CO	*Cercopithecidae*	*Cercopithecus campbelli*	M_Africa	F	LC	NI	DI	AR	M	N_FS	FRU	5	92
CO	*Cercopithecidae*	*Cercopithecus mitis*	M_Africa	F	LC	D	DI	AR	M	FS	O	10	186
CO	*Cercopithecidae*	*Cercopithecus mitis*	M_Africa	TR	LC	D	DI	AR	M	FS	O	10	28
CO	*Cheirogaleidae*	*Cheirogaleus major*	Madagascar	TR	DD	D	N	AR	S	FS	F_F	2	145
CO	*Pitheciidae*	*Chiropotes chiropotes*	Neotropics	TR	LC	S	DI	AR	M	FS	FRU	2	27
CO	*Pitheciidae*	*Chiropotes satanas*	Neotropics	TR	CR	D	DI	AR	M	FS	FRU	2	26
CO	*Cercopithecidae*	*Chlorocebus aethiops*	M_Africa	R	LC	S	DI	T	M	N_FS	O	12	235

CO	*Cercopithecidae*	*Colobus angolensis*	M_Africa	TR	LC	NI	DI	AR	M	FS	FOL	5	6
CO	*Lemuridae*	*Eulemur fulvus*	Madagascar	TR	NT	D	CATH	AR	M	FS	F_F	2	145
CO	*Lemuridae*	*Eulemur rubriventer*	Madagascar	TR	VU	D	CATH	AR	S	FS	F_F	1	145
CO	*Lemuridae*	*Hapalemur griseus*	Madagascar	TR	VU	D	DI	AR	S	FS	FOL	2	145
CO	*Lemuridae*	*Hapalemur meridionalis*	Madagascar	ALL	VU	D	CATH	AR	S	FS	FOL	1	65
CO	*Hylobatidae*	*Hoolock hoolock*	Asia	TR	EN	D	DI	AR	M	FS	FRU	1	52
CO	*Hylobatidae*	*Hylobates agilis*	Asia	F	EN	D	DI	AR	M	FS	FRU	1	167
CO	*Callitrichidae*	*Leontopithecus chrysomelas*	Neotropics	ALL	EN	D	DI	AR	S	FS	O	4	201
CO	*Callitrichidae*	*Leontopithecus chrysopygus*	Neotropics	TR	EN	D	DI	AR	S	FS	FRU	1	244
CO	*Cercopithecidae*	*Macaca fascicularis*	Asia	F	LC	D	DI	AR	M	FS	FRU	10	167
CO	*Cheirogaleidae*	*Microcebus murinus*	Madagascar	TR	LC	D	N	AR	S	N_FS	O	3	8
CO	*Hylobatidae*	*Nomascus concolor*	Asia	F	CR	D	DI	AR	M	FS	F_F	2	189
CO	*Hominidae*	*Pan troglodytes*	M_Africa	F	EN	D	DI	BOTH	L	N_FS	O	17	153
CO	*Cercopithecidae*	*Papio anubis*	M_Africa	ALL	LC	I	DI	T	L	N_FS	O	11	214
CO	*Cercopithecidae*	*Papio hamadryas*	M_Africa	TR	LC	I	DI	T	L	N_FS	O	4	188
CO	*Cercopithecidae*	*Piliocolobus badius*	M_Africa	R	EN	D	DI	AR	M	FS	F_F	5	84
CO	*Pitheciidae*	*Plecturocebus cupreus*	Neotropics	TR	LC	NI	DI	AR	S	FS	FRU	2	194
CO	*Pitheciidae*	*Plecturocebus Moloch*	Neotropics	NR	LC	NI	DI	AR	S	FS	O	2	130
CO	*Pitheciidae*	*Plecturocebus oenanthe*	Neotropics	F	CR	D	DI	AR	S	N_FS	O	1	66
CO	*Pitheciidae*	*Plecturocebus olallae*	Neotropics	NR	EN	D	DI	AR	S	N_FS	F_F	2	76
CO	*Cercopithecidae*	*Presbytis melalophos*	Asia	F	NT	D	DI	AR	M	N_FS	F_F	2	167
CO	*Indriidae*	*Propithecus diadema*	Madagascar	TR	CR	D	DI	AR	M	FS	F_F	1	145
CO	*Cercopithecidae*	*Rhinopithecus bieti*	Asia	TR	EN	D	DI	BOTH	L	FS	FOL	2	134
CO	*Cercopithecidae*	*Rungwecebus kipunji*	M_Africa	TR	CR	D	DI	AR	L	FS	O	2	28
CO	*Callitrichidae*	*Saguinus leucopus*	Neotropics	TR	EN	D	DI	AR	S	FS	FRU	7	209
CO	*Callitrichidae*	*Saguinus leucopus*	Neotropics	F	EN	D	DI	AR	S	FS	FRU	7	53
CO	*Callitrichidae*	*Saguinus leucopus*	Neotropics	TR	EN	D	DI	AR	S	FS	FRU	7	127
CO	*Cebidae*	*Saimiri cassiquiarensis*	Neotropics	NR	LC	NI	DI	AR	S	N_FS	O	1	35
CO	*Cebidae*	*Saimiri oerstedii*	Neotropics	NR	VU	D	DI	AR	S	FS	O	3	69
CO	*Cebidae*	*Saimiri sciureus*	Neotropics	F	LC	D	DI	AR	S	FS	O	3	194
CO	*Cebidae*	*Sapajus apella*	Neotropics	F	LC	D	DI	AR	M	N_FS	O	11	194

CO	*Cebidae*	*Sapajus apella*	Neotropics	NR	LC	D	DI	AR	M	N_FS	O	11	130
CO	*Cebidae*	*Sapajus apella*	Neotropics	TR	LC	D	DI	AR	M	N_FS	O	11	244
CO	*Cebidae*	*Sapajus libidinosus*	Neotropics	ALL	LC	D	DI	AR	M	N_FS	O	3	2
CO	*Cebidae*	*Sapajus nigritus*	Neotropics	ALL	NT	D	DI	AR	M	FS	O	4	2
CO	*Cercopithecidae*	*Semnopithecus vetulus*	Asia	TR	EN	D	DI	AR	M	FS	F_F	10	211
CO	*Cercopithecidae*	*Semnopithecus vetulus*	Asia	TR	EN	D	DI	AR	M	FS	F_F	10	163
CO	*Cercopithecidae*	*Semnopithecus vetulus*	Asia	TR	EN	D	DI	AR	M	FS	F_F	10	182
CO	*Cercopithecidae*	*Semnopithecus vetulus*	Asia	F	EN	D	DI	AR	M	FS	F_F	10	56
SF	*Cercopithecidae*	*Allochrocebus lhoesti*	M_Africa	NR	VU	D	DI	T	M	N_FS	O	2	140
SF	*Cercopithecidae*	*Allochrocebus lhoesti*	M_Africa	ALL	VU	D	DI	T	M	N_FS	O	2	118
SF	*Atelidae*	*Alouatta belzebul*	Neotropics	NR	VU	D	DI	AR	M	FS	F_F	1	246
SF	*Atelidae*	*Alouatta guariba*	Neotropics	NR	LC	D	DI	AR	M	FS	F_F	5	193
SF	*Atelidae*	*Alouatta guariba*	Neotropics	ALL	LC	D	DI	AR	M	FS	F_F	5	116
SF	*Atelidae*	*Alouatta macconnelli*	Neotropics	NR	LC	NI	DI	AR	M	N_FS	F_F	3	133
SF	*Atelidae*	*Alouatta macconnelli*	Neotropics	NR	LC	NI	DI	AR	M	N_FS	F_F	3	184
SF	*Atelidae*	*Alouatta macconnelli*	Neotropics	NR	LC	NI	DI	AR	M	N_FS	F_F	3	183
SF	*Atelidae*	*Alouatta palliata*	Neotropics	NR	LC	NI	DI	AR	M	FS	F_F	25	181
SF	*Atelidae*	*Alouatta palliata*	Neotropics	NR	LC	NI	DI	AR	M	FS	F_F	25	230
SF	*Atelidae*	*Alouatta palliata*	Neotropics	TR	LC	NI	DI	AR	M	FS	F_F	25	74
SF	*Atelidae*	*Alouatta palliata*	Neotropics	NR	LC	NI	DI	AR	M	FS	F_F	25	54
SF	*Atelidae*	*Alouatta palliata*	Neotropics	NR	LC	NI	DI	AR	M	FS	F_F	25	40
SF	*Atelidae*	*Alouatta palliata*	Neotropics	NR	LC	NI	DI	AR	M	FS	F_F	25	41
SF	*Atelidae*	*Alouatta pigra*	Neotropics	NR	EN	D	DI	AR	M	FS	F_F	12	242
SF	*Atelidae*	*Alouatta seniculus*	Neotropics	ALL	LC	NI	DI	AR	M	N_FS	F_F	7	98
SF	*Atelidae*	*Alouatta seniculus*	Neotropics	NR	LC	NI	DI	AR	M	N_FS	F_F	7	19
SF	*Lorisidae*	*Arctocebus calabarensis*	M_Africa	NR	LC	NI	N	AR	S	FS	IN	1	88
SF	*Atelidae*	*Ateles belzebuth*	Neotropics	ALL	EN	D	DI	AR	M	FS	FRU	2	98
SF	*Atelidae*	*Ateles geoffroyi*	Neotropics	NR	EN	D	DI	AR	M	FS	FRU	19	230
SF	*Atelidae*	*Ateles geoffroyi*	Neotropics	NR	EN	D	DI	AR	M	FS	FRU	19	54
SF	*Atelidae*	*Ateles geoffroyi*	Neotropics	F	EN	D	DI	AR	M	FS	FRU	19	203
SF	*Atelidae*	*Ateles geoffroyi*	Neotropics	NR	EN	D	DI	AR	M	FS	FRU	19	42

SF	*Atelidae*	*Ateles geoffroyi*	Neotropics	NR	EN	D	DI	AR	M	FS	FRU	19	242
SF	*Atelidae*	*Ateles geoffroyi*	Neotropics	TR	EN	D	DI	AR	M	FS	FRU	19	204
SF	*Atelidae*	*Ateles geoffroyi*	Neotropics	F	EN	D	DI	AR	M	FS	FRU	19	205
SF	*Atelidae*	*Ateles geoffroyi*	Neotropics	NR	EN	D	DI	AR	M	FS	FRU	19	41
SF	*Atelidae*	*Ateles paniscus*	Neotropics	NR	VU	D	DI	AR	M	FS	FRU	2	133
SF	*Atelidae*	*Ateles paniscus*	Neotropics	NR	VU	D	DI	AR	M	FS	FRU	2	184
SF	*Pitheciidae*	*Callicebus coimbrai*	Neotropics	NR	EN	D	DI	AR	S	N_FS	FRU	1	39
SF	*Pitheciidae*	*Callicebus melanochir*	Neotropics	NR	VU	D	DI	AR	S	FS	FRU	1	80
SF	*Pitheciidae*	*Callicebus personatus*	Neotropics	NR	VU	D	DI	AR	S	FS	FRU	2	193
SF	*Pitheciidae*	*Callicebus personatus*	Neotropics	ALL	VU	D	DI	AR	S	FS	FRU	2	103
SF	*Callitrichidae*	*Callithrix jacchus*	Neotropics	NR	LC	S	DI	AR	S	N_FS	G	2	39
SF	*Callitrichidae*	*Callithrix penicillata*	Neotropics	NR	LC	I	DI	AR	S	N_FS	G	7	80
SF	*Cebidae*	*Cebus albifrons*	Neotropics	ALL	LC	D	DI	BOTH	M	FS	O	2	98
SF	*Cebidae*	*Cebus albifrons*	Neotropics	NR	LC	D	DI	BOTH	M	FS	O	2	19
SF	*Cebidae*	*Cebus capucinus*	Neotropics	NR	LC	NI	DI	AR	M	FS	O	15	230
SF	*Cebidae*	*Cebus capucinus*	Neotropics	NR	LC	NI	DI	AR	M	FS	O	15	54
SF	*Cebidae*	*Cebus capucinus*	Neotropics	ALL	LC	NI	DI	AR	M	FS	O	15	43
SF	*Cebidae*	*Cebus capucinus*	Neotropics	NR	LC	NI	DI	AR	M	FS	O	15	41
SF	*Cercopithecidae*	*Cercocebus atys*	M_Africa	NR	NT	D	DI	T	M	N_FS	FRU	2	79
SF	*Cercopithecidae*	*Cercocebus atys*	M_Africa	NR	NT	D	DI	T	M	N_FS	FRU	2	78
SF	*Cercopithecidae*	*Cercocebus galeritus*	M_Africa	F	EN	D	DI	BOTH	M	FS	FRU	3	251
SF	*Cercopithecidae*	*Cercopithecus ascanius*	M_Africa	NR	LC	NI	DI	AR	M	FS	FRU	12	239
SF	*Cercopithecidae*	*Cercopithecus ascanius*	M_Africa	F	LC	NI	DI	AR	M	FS	FRU	12	223
SF	*Cercopithecidae*	*Cercopithecus ascanius*	M_Africa	NR	LC	NI	DI	AR	M	FS	FRU	12	140
SF	*Cercopithecidae*	*Cercopithecus campbelli*	M_Africa	NR	LC	NI	DI	AR	M	N_FS	FRU	5	79
SF	*Cercopithecidae*	*Cercopithecus campbelli*	M_Africa	NR	LC	NI	DI	AR	M	N_FS	FRU	5	78
SF	*Cercopithecidae*	*Cercopithecus cephus*	M_Africa	NR	LC	NI	DI	AR	M	FS	FRU	2	88
SF	*Cercopithecidae*	*Cercopithecus diana*	M_Africa	NR	VU	D	DI	AR	M	FS	FRU	2	79
SF	*Cercopithecidae*	*Cercopithecus diana*	M_Africa	NR	VU	D	DI	AR	M	FS	FRU	2	78
SF	*Cercopithecidae*	*Cercopithecus mitis*	M_Africa	NR	LC	D	DI	AR	M	FS	O	10	239
SF	*Cercopithecidae*	*Cercopithecus mitis*	M_Africa	F	LC	D	DI	AR	M	FS	O	10	118

SF	*Cercopithecidae*	*Cercopithecus nictitans*	M_Africa	NR	LC	D	DI	AR	M	FS	FRU	3	88
SF	*Cercopithecidae*	*Cercopithecus petaurista*	M_Africa	NR	LC	NI	DI	AR	M	N_FS	O	2	79
SF	*Cercopithecidae*	*Cercopithecus petaurista*	M_Africa	NR	LC	NI	DI	AR	M	N_FS	O	2	78
SF	*Cercopithecidae*	*Cercopithecus pogonias*	M_Africa	NR	LC	D	DI	AR	M	N_FS	FRU	3	88
SF	*Cercopithecidae*	*Cercopithecus pogonias*	M_Africa	NR	LC	D	DI	AR	M	N_FS	FRU	3	239
SF	*Pitheciidae*	*Chiropotes chiropotes*	Neotropics	NR	LC	S	DI	AR	M	FS	FRU	2	133
SF	*Pitheciidae*	*Chiropotes satanas*	Neotropics	TR	CR	D	DI	AR	M	FS	FRU	2	90
SF	*Cercopithecidae*	*Colobus angolensis*	M_Africa	NR	LC	NI	DI	AR	M	FS	FOL	5	239
SF	*Cercopithecidae*	*Colobus guereza*	M_Africa	NR	LC	NI	DI	AR	M	FS	F_F	3	239
SF	*Cercopithecidae*	*Colobus guereza*	M_Africa	NR	LC	NI	DI	AR	M	FS	F_F	3	140
SF	*Cercopithecidae*	*Colobus guereza*	M_Africa	F	LC	NI	DI	AR	M	FS	F_F	3	71
SF	*Cercopithecidae*	*Colobus polykomos*	M_Africa	NR	VU	NI	DI	AR	M	FS	FOL	2	79
SF	*Cercopithecidae*	*Colobus polykomos*	M_Africa	NR	VU	NI	DI	AR	M	FS	FOL	2	78
SF	*Daubentoniidae*	*Daubentonia madagascariensis*	Madagascar	ALL	EN	D	N	AR	M	N_FS	IN	1	5
SF	*Lemuridae*	*Eulemur flavifrons*	Madagascar	ALL	CR	D	CATH	AR	S	FS	F_F	1	224
SF	*Lemuridae*	*Eulemur macaco*	Madagascar	ALL	VU	D	CATH	AR	M	FS	FRU	1	16
SF	*Galagidae*	*Galago elegantulus*	M_Africa	NR	LC	S	N	AR	S	FS	G	1	88
SF	*Galagidae*	*Galagoides demidovii*	M_Africa	NR	LC	S	N	AR	S	FS	O	1	88
SF	*Hominidae*	*Gorilla beringei*	M_Africa	F	EN	D	DI	BOTH	L	FS	F_F	1	257
SF	*Hominidae*	*Gorilla gorilla*	M_Africa	R	CR	D	DI	BOTH	L	FS	F_F	2	88
SF	*Hominidae*	*Gorilla gorilla*	M_Africa	R	CR	D	DI	BOTH	L	FS	F_F	2	148
SF	*Hylobatidae*	*Hylobates lar*	Asia	NR	EN	D	DI	AR	M	FS	FRU	2	115
SF	*Hylobatidae*	*Hylobates muelleri*	Asia	NR	EN	D	DI	AR	M	FS	FRU	1	91
SF	*Callitrichidae*	*Leontocebus fuscicollis*	Neotropics	F	LC	D	DI	AR	S	FS	O	4	50
SF	*Callitrichidae*	*Leontocebus fuscicollis*	Neotropics	F	LC	D	DI	AR	S	FS	O	4	51
SF	*Callitrichidae*	*Leontocebus fuscicollis*	Neotropics	R	LC	D	DI	AR	S	FS	O	4	129
SF	*Callitrichidae*	*Leontocebus nigrifrons*	Neotropics	F	LC	D	DI	AR	S	FS	O	1	126
SF	*Cercopithecidae*	*Lophocebus albigena*	M_Africa	NR	LC	D	DI	AR	M	FS	FRU	2	239
SF	*Cercopithecidae*	*Lophocebus albigena*	M_Africa	NR	LC	D	DI	AR	M	FS	FRU	2	140
SF	*Cercopithecidae*	*Macaca fascicularis*	Asia	NR	LC	D	DI	AR	M	FS	FRU	10	115
SF	*Cercopithecidae*	*Macaca nemestrina*	Asia	NR	VU	D	DI	T	M	FS	FRU	4	115

SF	*Cercopithecidae*	*Macaca nigra*	Asia	F	CR	D	DI	T	M	FS	FRU	1	212
SF	*Cercopithecidae*	*Mandrillus sphinx*	M_Africa	ALL	VU	NI	DI	T	L	FS	O	1	88
SF	*Cheirogaleidae*	*Microcebus murinus*	Madagascar	NR	LC	D	N	AR	S	N_FS	O	3	87
SF	*Cercopithecidae*	*Miopithecus talapoin*	M_Africa	NR	LC	NI	DI	BOTH	S	FS	O	2	88
SF	*Hylobatidae*	*Nomascus concolor*	Asia	ALL	CR	D	DI	AR	M	FS	F_F	2	189
SF	*Hominidae*	*Pan troglodytes*	M_Africa	F	EN	D	DI	BOTH	L	N_FS	O	17	14
SF	*Hominidae*	*Pan troglodytes*	M_Africa	R	EN	D	DI	BOTH	L	N_FS	O	17	88
SF	*Hominidae*	*Pan troglodytes*	M_Africa	NR	EN	D	DI	BOTH	L	N_FS	O	17	140
SF	*Cercopithecidae*	*Papio anubis*	M_Africa	NR	LC	I	DI	T	L	N_FS	O	11	140
SF	*Lorisidae*	*Perodicticus potto*	M_Africa	NR	LC	S	N	AR	S	FS	FRU	1	88
SF	*Cercopithecidae*	*Piliocolobus badius*	M_Africa	NR	EN	D	DI	AR	M	FS	F_F	5	239
SF	*Cercopithecidae*	*Piliocolobus badius*	M_Africa	NR	EN	D	DI	AR	M	FS	F_F	5	79
SF	*Cercopithecidae*	*Piliocolobus badius*	M_Africa	NR	EN	D	DI	AR	M	FS	F_F	5	78
SF	*Cercopithecidae*	*Piliocolobus tephrosceles*	M_Africa	NR	EN	D	DI	AR	M	FS	FOL	1	140
SF	*Pitheciidae*	*Pithecia irrorata*	Neotropics	NR	LC	NI	DI	AR	M	FS	FRU	1	246
SF	*Pitheciidae*	*Pithecia pithecia*	Neotropics	NR	LC	NI	DI	AR	S	FS	FRU	2	133
SF	*Pitheciidae*	*Pithecia pithecia*	Neotropics	NR	LC	NI	DI	AR	S	FS	FRU	2	183
SF	*Pitheciidae*	*Plecturocebus cupreus*	Neotropics	ALL	LC	NI	DI	AR	S	FS	FRU	2	123
SF	*Hominidae*	*Pongo pygmaeus*	Asia	ALL	EN	D	DI	AR	L	FS	FRU	2	216
SF	*Cercopithecidae*	*Presbytis hosei*	Asia	NR	DD	D	DI	AR	M	FS	FOL	1	171
SF	*Cercopithecidae*	*Presbytis melalophos*	Asia	NR	NT	D	DI	AR	M	N_FS	F_F	2	115
SF	*Cercopithecidae*	*Procolobus verus*	M_Africa	NR	NT	NI	DI	AR	M	FS	FOL	2	79
SF	*Cercopithecidae*	*Procolobus verus*	M_Africa	ALL	NT	NI	DI	AR	M	FS	FOL	2	174
SF	*Indriidae*	*Propithecus verreauxi*	Madagascar	ALL	EN	D	DI	AR	M	N_FS	F_F	4	172
SF	*Cercopithecidae*	*Rhinopithecus bieti*	Asia	F	EN	D	DI	BOTH	L	FS	FOL	2	255
SF	*Cercopithecidae*	*Rhinopithecus roxellana*	Asia	NR	EN	D	DI	BOTH	L	FS	FOL	1	135
SF	*Callitrichidae*	*Saguinus leucopus*	Neotropics	ALL	EN	D	DI	AR	S	FS	FRU	7	98
SF	*Callitrichidae*	*Saguinus leucopus*	Neotropics	ALL	EN	D	DI	AR	S	FS	FRU	7	19
SF	*Callitrichidae*	*Saguinus midas*	Neotropics	NR	LC	S	DI	AR	S	N_FS	O	2	133
SF	*Callitrichidae*	*Saguinus midas*	Neotropics	NR	LC	S	DI	AR	S	N_FS	O	2	183
SF	*Callitrichidae*	*Saguinus mystax*	Neotropics	F	LC	S	DI	AR	S	FS	O	4	50

SF	*Callitrichidae*	*Saguinus mystax*	Neotropics	F	LC	S	DI	AR	S	FS	O	4	51
SF	*Callitrichidae*	*Saguinus mystax*	Neotropics	R	LC	S	DI	AR	S	FS	O	4	129
SF	*Cebidae*	*Saimiri oerstedii*	Neotropics	ALL	VU	D	DI	AR	S	FS	O	3	24
SF	*Cebidae*	*Saimiri sciureus*	Neotropics	NR	LC	D	DI	AR	S	FS	O	3	183
SF	*Cebidae*	*Sapajus apella*	Neotropics	NR	LC	D	DI	AR	M	N_FS	O	11	133
SF	*Cebidae*	*Sapajus apella*	Neotropics	NR	LC	D	DI	AR	M	N_FS	O	11	184
SF	*Cebidae*	*Sapajus apella*	Neotropics	NR	LC	D	DI	AR	M	N_FS	O	11	193
SF	*Cebidae*	*Sapajus apella*	Neotropics	NR	LC	D	DI	AR	M	N_FS	O	11	183
SF	*Cebidae*	*Sapajus apella*	Neotropics	TR	LC	D	DI	AR	M	N_FS	O	11	90
SF	*Cebidae*	*Sapajus apella*	Neotropics	NR	LC	D	DI	AR	M	N_FS	O	11	246
SF	*Cebidae*	*Sapajus xanthosternos*	Neotropics	NR	CR	D	DI	AR	M	FS	O	4	80
SF	*Cebidae*	*Sapajus xanthosternos*	Neotropics	NR	CR	D	DI	AR	M	FS	O	4	39
SF	*Cebidae*	*Sapajus xanthosternos*	Neotropics	F	CR	D	DI	AR	M	FS	O	4	33
SF	*Galagidae*	*Sciurocheirus alleni*	M_Africa	NR	EN	NI	N	AR	S	FS	FRU	1	88
SF	*Cercopithecidae*	*Trachypithecus auratus*	Asia	ALL	VU	D	DI	AR	M	FS	FOL	2	122
SF	*Cercopithecidae*	*Trachypithecus obscurus*	Asia	NR	NT	D	DI	AR	M	FS	FOL	2	115
SF	*Cercopithecidae*	*Trachypithecus phayrei*	Asia	ALL	EN	D	DI	AR	M	N_FS	FOL	1	99
SF	*Lemuridae*	*Varecia rubra*	Madagascar	F	CR	D	DI	AR	M	FS	FRU	1	144

[a]For taxonomy, we followed: Estrada, A. *et al*. Impending extinction crisis of the world's primates: why primates matter. *Sci. Adv.* **3,** e1600946 (2017).

Supplementary Table 2. Sensitivity analysis for phylogenetic signal in matrix use. Each row shows the focal family with its number of species (N), the estimate of D obtained after removing it, the % change from the value for the whole order, and the results from the randomization test. m.null.estimate is the mean value of the null distribution of estimates after 500 randomizations. Pval.randomization is the result of testing if the change in parameter estimate is significantly different from the null distribution.

Clade removed	N	D	Percent change	m.null.estimate	Pval.randomization
Lepilemuridae	24	0.881	5.7	0.832	0.025
Cheirogaleidae	28	0.859	3.1	0.830	0.135
Atelidae	22	0.853	2.5	0.832	0.165
Pitheciidae	18	0.813	2.4	0.831	0.190
Hylobatidae	18	0.815	2.1	0.831	0.170
Callitrichidae	29	0.823	1.2	0.833	0.328
Galagidae	14	0.825	0.9	0.831	0.350
Cercopithecidae	120	0.827	0.8	0.827	0.495
Lemuridae	21	0.826	0.8	0.830	0.415
Indriidae	15	0.830	0.4	0.831	0.455
Cebidae	12	0.830	0.3	0.831	0.463

Supplementary References

1. Agoramoorthy, G. in *Faunal ecology and conservation of the great Indian desert* (Sivaperuman, C., Baqri, Q. H., Ramaswamy, G. & Naseema, M. eds.) 177–191 (Springer, 2009).

2. Aguiar, L. M. *et al.* Occurrence, local extinction and conservation of primates in the corridor of the Upper Paraná River, with notes on other mammals. *Rev. Bras. Zool.* **24,** 898–906 (2007).

3. Albert, A., Savini, T. & Huynen, M. C. Sleeping site selection and presleep behavior in wild pigtailed macaques. *Am. J. Primatol.* **73,** 1222–1230 (2011).

4. Altmann, J. & Muruthi, P. Differences in daily life between semiprovisioned and wild-feeding baboons. *Am. J. Primatol.* **15,** 213–221 (1988).

5. Ancrenaz, M., Lackman-Ancrenaz, I. & Mundy, N. Field observations of aye-ayes (*Daubentonia madagascariensis*) in Madagascar. *Folia Primatol.* **62,** 22–36 (1994).

6. Anderson, J., Rowcliffe, J. M. & Cowlishaw, G. Does the matrix matter? A forest primate in a complex agricultural landscape. *Biol. Conserv.* **135,** 212–222 (2007).

7. Anderson, J., Rowcliffe, J. M. & Cowlishaw, G. The Angola black-and-white colobus (*Colobus angolensis palliatus*) in Kenya: historical range contraction and current conservation status. *Am. J. Primatol.* **69,** 664–680 (2007).

8. Andriamandimbiarisoa, L. *et al.* Habitat corridor utilization by the gray mouse lemur, *Microcebus murinus*, in the littoral forest fragments of southeastern Madagascar. *Madag. Conserv. Dev.* **10,** 144–150 (2015).

9. Aristizábal-Borja, J., Pozo-Montuy, G., Pérez-Torres, J. & Serio-Silva, J. C. Anotaciones de la ecología alimentaria de monos aulladores negros en un fragmento con condiciones de hacinamiento (Balancán, Tabasco, México). *Univ. Sci.* **16,** 140–146 (2011).

10. Asensio, N., Arroyo-Rodríguez, V., Dunn, J. C. & Cristóbal-Azkarate, J. Conservation value of landscape supplementation for howler monkeys living in forest patches. *Biotropica* **41,** 768–773 (2009).

11. Azhar, B., Lindenmayer, D. B., Wood, J., Fischer, J. & Zakaria, M. Ecological impacts of oil palm agriculture on forest mammals in plantation estates and smallholdings. *Biodivers. Conserv.* **23,** 1175–1191 (2014).

12. Baker, L. R., Tanimola, A. A. & Olubode, O. S. Sacred populations of *Cercopithecus sclateri*: analysis of apparent population increases from census counts. *Am. J. Primatol.* **76,** 303–312 (2014).

13. Baranga, D., Basuta, G. I., Teichroeb, J. A. & Chapman, C. A. Crop raiding patterns of solitary and social groups of red-tailed monkeys on cocoa pods in Uganda. *Trop. Conserv. Sci.* **5,** 104–111 (2012).

14. Basabose, A. K. Ranging patterns of chimpanzees in a montane forest of Kahuzi,

Democratic Republic of Congo. *Int. J. Primatol.* **26,** 33–54 (2005).

15. Baya, L. & Storch, I. Status of diurnal primate populations at the former settlement of a displaced village in Cameroon. *Am. J. Primatol.* **72,** 645–652 (2010).

16. Bayart, F. & Simmen, B. Demography, range use, and behavior in black lemurs (*Eulemur macaco macaco*) at Ampasikely, northwest Madagascar. *Am. J. Primatol.* **67,** 299–312 (2005).

17. Beisner, B. A. *et al.* Human–wildlife conflict: proximate predictors of aggression between humans and rhesus macaques in India. *Am. J. Phys. Anthropol.* **156,** 286–294 (2015).

18. Benitez-Malvido, J. *et al.* The role of sex and age in the architecture of intrapopulation howler monkey-plant networks in continuous and fragmented rain forests. *PeerJ* **4,** e1809 (2016).

19. Bernstein, I. S. *et al.* Differential effects of forest degradation on primate populations. *Primates* **17,** 401–411 (1976).

20. Bessa, J., Sousa, C. & Hockings, K. J. Feeding ecology of chimpanzees (*Pan troglodytes verus*) inhabiting a forest-mangrove-savanna-agricultural matrix at Caiquene-Cadique, Cantanhez National Park, Guinea-Bissau. *Am. J. Primatol.* **77,** 651–665 (2015).

21. Bhat, S. K., Nair, C. P. R. & Mathew, D. N. Mammalian pests of cocoa in South India. *Int. J. Pest Manage.* **27,** 297–302 (1981).

22. Bicca-Marques, J. C. & Calegaro-Marques, C. Locomotion of black howlers in a habitat with discontinuous canopy. *Folia Primatol.* **64,** 55–61 (1995).

23. Bismark, M. in *Indonesian Primates* (Gursky-Doyen, S. & Supriatna, J. eds.) 217–233 (Springer, 2010).

24. Blair, M. E. & Melnick, D. J. Scale-dependent effects of a heterogeneous landscape on genetic differentiation in the Central American squirrel monkey (*Saimiri oerstedii*). *PLoS One* **7,** e43027 (2012).

25. Boulton, A. M., Horrocks, J. A. & Baulu, J. The barbados vervet monkey (*Cercopithecus aethiops sabaens*): changes in population size and crop damage, 1980–1994. *Int. J. Primatol.* **17,** 831–844 (1996).

26. Boyle, S. A. & Smith, A. T. Behavioral modifications in northern bearded saki monkeys (*Chiropotes satanas chiropotes*) in forest fragments of central Amazonia. *Primates* **51,** 43 (2010).

27. Boyle, S. A., Zartman, C. E., Spironello, W. R. & Smith, A. T. Implications of habitat fragmentation on the diet of bearded saki monkeys in central Amazonian forest. *J. Mammal.* **93,** 959–976 (2012).

28. Bracebridge, C. E., Davenport, T. R., Mbofu, V. F. & Marsden, S. J. Is there a role for human-dominated landscapes in the long-term conservation management of the

critically endangered kipunji (*Rungwecebus kipunji*)? *Int. J. Primatol.* **34,** 1122–1136 (2013).

29. Braza, F., Alvarez, F. & Azcarate, T. Behaviour of the red howler monkey (*Alouatta seniculus*) in the Llanos of Venezuela. *Primates* **22,** 459–473 (1981).

30. Brotcorne, F. *et al.* The role of anthropic, ecological, and social factors in sleeping site choice by long-tailed macaques (*Macaca fascicularis*). *Am. J. Primatol.* **76,** 1140–1150 (2014).

31. Campbell-Smith, G., Campbell-Smith, M., Singleton, I. & Linkie, M. Raiders of the lost bark: orangutan foraging strategies in a degraded landscape. *PLoS One* **6,** e20962 (2011).

32. Canale, G. R. *et al.* Seed dispersal of threatened tree species by a critically endangered primate in a Brazilian hotspot. *Folia Primatol.* **87,** 123–140 (2016).

33. Canale, G. R., Kierulff, M. C. M. & Chivers, D. J. in *Primates in fragments* (Marsh, L. K. & Chapman, C. A. eds.) 299–311 (Springer, 2013).

34. Carretero-Pinzón, X. in *Primates in fragments* (Marsh, L. K. & Chapman, C. A. eds.) 159–182 (Springer, 2013).

35. Carretero-Pinzón, X., Defler, T. R. & Ruiz-Garcia, M. in *Phylogeny, molecular population genetics, evolutionary biology and conservation of the Neotropical primates* (J., M. R.-G. & Shostell, M. eds.) 491–506 (Nova Science Publisher, 2016).

36. Carvalho, J. S., Marques, T. A. & Vicente, L. Population status of *Pan troglodytes verus* in Lagoas de Cufada Natural Park, Guinea-Bissau. *PLoS One* **8,** e71527 (2013).

37. Carvalho, J. S., Vicente, L. & Marques, T. A. Chimpanzee (*Pan troglodytes verus*) diet composition and food availability in a human-modified landscape at Lagoas de Cufada natural Park, Guinea-Bissau. *Int. J. Primatol.* **36,** 802–822 (2015).

38. Castaño, J. H., Ramírez, D. C. & Botero, J. E. in *Primatología en Colombia: avances al principio del milenio* (Pereira-Bengoa, V., Stevenson, P. R., Bueno, M. L. & Nassar-Montoya, F., eds.) 69–90 (Fundación Universitaria San Martín, 2010).

39. Chagas, R. R. D. & Ferrari, S. F. Habitat use by *Callicebus coimbrai* (Primates: Pitheciidae) and sympatric species in the fragmented landscape of the Atlantic Forest of southern Sergipe, Brazil. *Zool.* **27,** 853–860 (2010).

40. Chapman, C. A. & Balcomb, S. R. Population characteristics of howlers: ecological conditions or group history. *Int. J. Primatol.* **19,** 385–403 (1998).

41. Chapman, C. A. & Fedigan, L. M. Dietary differences between neighboring *Cebus capucinus* groups: local traditions, food availability or responses to food profitability? *Folia Primatol.* **54,** 177–186 (1990).

42. Chapman, C. A. & Onderdonk, D. A. Forests without primates: primate/plant codependency. *Am. J. Primatol.* **45,** 127–141 (1998).

43. Chapman, C. A., Chapman, L. & Glander, K. E. Primate populations in northwestern Costa Rica: potential for recovery. *Primate Conserv.* **10,** 37–44 (1989).

44. Chapman, C. A., Chapman, L. J. & Wrangham, R. W. Ecological constraints on group size: an analysis of spider monkey and chimpanzee subgroups. *Behav. Ecol. Sociobiol.* **36,** 59–70 (1995).

45. Ciani, A. C. & Chiarelli, B. Age and sex differences in the feeding strategies of a free-ranging population of *Macaca mulatta* Zimmerman, 1788 (primates: Cercopithecidae), in Simla (India). *Monit. Zool. Ital. J. Zool.* **22,** 171–182 (1988).

46. Ciani, A. C. Intertroop agonistic behavior of a feral rhesus macaque troop ranging in town and forest areas in India. *Aggressive Behav.* **12,** 433–439 (1986).

47. Coss, R. G. & Ramakrishnan, U. Perceptual aspects of leopard recognition by wild bonnet macaques (*Macaca radiata*). *Behaviour* **137,** 315–335 (2000).

48. Coss, R. G., Marks, S. & Ramakrishnan, U. Early environment shapes the development of gaze aversion by wild bonnet macaques (*Macaca radiata*). *Primates* **43,** 217–222 (2002).

49. Coss, R. G., McCowan, B. & Ramakrishnan, U. Threat-related acoustical differences in alarm calls by wild bonnet macaques (*Macaca radiata*) elicited by python and leopard models. *Ethology* **113,** 352–367 (2007).

50. Culot, L., Lazo, F. J. J. M., Huynen, M. C., Poncin, P. & Heymann, E. W. Seasonal variation in seed dispersal by tamarins alters seed rain in a secondary rain forest. *Int. J. Primatol.* **31,** 553–569 (2010).

51. Culot, L., Mann, D. J., Muñoz Lazo, F. J., Huynen, M. C. & Heymann, E. W. Tamarins and dung beetles: an efficient diplochorous dispersal system in the Peruvian Amazonia. *Biotropica* **43,** 84–92 (2011).

52. Das, J., Biswas, J., Bhattacherjee, P. C. & Rao, S. S. in *The gibbons. Developments in primatology: progress and prospects* (Whittaker, D. & Lappan, S. eds.) 467–475 (Springer, 2009).

53. de Luna, G. A., García-Morera, Y. & Link, A. Behavior and ecology of the white-footed tamarin (*Saguinus leucopus*) in a fragmented landscape of Colombia: small bodied primates and seed dispersal in Neotropical forests. *Trop. Conserv. Sci.* **9,** 788–808 (2016).

54. DeGama-Blanchet, H. N. & Fedigan, L. M. in *New perspectives in the study of mesoamerican primates* (Estrada, A., Garber, P. A., Pavelka, M. M. & Luecke, L. eds.) 165–188 (Springer, 2006).

55. Dela, J. D. Impact of monkey-human relationships and habitat change on *Semnopithecus vetulus nestor* in human modified habitats. *J. Natl. Sci. Found. Sri* **39,** 365–382 (2011).

56. Dela, J. D. Seasonal food use strategies of *Semnopithecus vetulus nestor*, at Panadura and Piliyandala, Sri Lanka. *Int. J. Primatol.* **28,** 607–626 (2007).

57. Dib, L. R. T., Oliva, A. S. & Strier, K. B. Terrestrial travel in muriquis (*Brachyteles arachnoides*) across a forest clearing at the Estacao Biologica de Caratinga, Minas Gerais, Brazil. *Neotrop. Primates* **5,** 8–9 (1997).

58. Dorgay, E., Muelle, R. & Klooster, A. *Exploring possibilities for reforestation in southwestern Nicaragua: the social and ecological dimensions of living fence rows*. 97 (2016).

59. Duarte, M. H. & Young, R. J. Sleeping site selection by urban marmosets (*Callithrix penicillata*) under conditions of exceptionally high predator density. *Int. J. Primatol.* **32,** 329–334 (2011).

60. Duarte, M. H., Goulart, V. D. & Young, R. J. Designing laboratory marmoset housing: what can we learn from urban marmosets? *Appl. Anim. Behav. Sci.* **137,** 127–136 (2012).

61. Duarte, M. H., Vecci, M. A., Hirsch, A. & Young, R. J. Noisy human neighbours affect where urban monkeys live. *Biol. Lett.* **7,** 840–842 (2011).

62. Duvall, C. S. Human settlement ecology and chimpanzee habitat selection in Mali. *Landscape Ecol.* **23,** 699 (2008).

63. Ekanayake, D. K., Rajapakse, R. P. V., Dubey, J. P. & Dittus, W. P. J. Seroprevalence of *Toxoplasma gondii* in wild toque macaques (*Macaca sinica*) at Polonnaruwa, Sri Lanka. *J. Parasitol.* **90,** 870–871 (2004).

64. Enari, H. & Sakamaki-Enari, H. Synergistic effects of primates and dung beetles on soil seed accumulation in snow regions. *Ecol. Res.* **29,** 653–660 (2014).

65. Eppley, T. M. *et al.* The use of an invasive species habitat by a small folivorous primate: implications for lemur conservation in Madagascar. *PLoS One* **10,** e0140981 (2015).

66. Ernest, M. M. Habitat quality and integrated connectivity analysis for *Callicebus oenanthe* in San Martin, Peru. (2015).

67. Eschmann, C., Moore, R. & Nekaris, K. A. I. Calling patterns of western purple-faced langurs (Mammalia Primates: Cercopithecidea: *Trachypithecus vetulus nestor*) in a degraded human landscape in Sri Lanka. *Contrib. Zool.* **77,** 57–65 (2008).

68. Estrada, A. & Coates-Estrada, R. Tropical rain forest fragmentation and wild populations of primates at Los Tuxtlas, Mexico. *Int. J. Primatol.* **17,** 759–783 (1996).

69. Estrada, A. *et al.* in *New perspectives in the study of Mesoamerican primates* (Estrada, A., Garber, P. A., Pavelka, M. M. & Luecke, L. eds.) 437–470 (Springer, 2006).

70. Fam, S. D., Lee, B. P. Y.-H. & Shekelle, M. The conservation status of slow lorises *Nycticebus* spp. in Singapore. *Endanger. Species Res.* **25,** 69–77 (2014).

71. Fashing, P. J. Mortality trends in the African cherry (*Prunus africana*) and the implications for colobus monkeys (*Colobus guereza*) in Kakamega Forest, Kenya. *Biol.*

Conserv. **120,** 449–459 (2004).

72. Fedigan, L. M. & Jack, K. Neotropical primates in a regenerating Costa Rican dry forest: a comparison of howler and capuchin population patterns. *Int. J. Primatol.* **22,** 689–713 (2001).

73. Fedigan, L. M., Fedigan, L., Chapman, C. A. & Glander, K. E. A census of *Alouatta palliata* and *Cebus capucinus* monkeys in Santa Rosa National Park, Costa Rica. *Brenesia.,* **23,** 309–322 (1985).

74. Fedigan, L. M., Rose, L. M. & Avila, R. M. Growth of mantled howler groups in a regenerating Costa Rican dry forest. *Int. J. Primatol.* **19,** 405–432 (1998).

75. Fehlmann, G., O'Riain, M. J., Kerr-Smith, C. & King, A. J. Adaptive space use by baboons (*Papio ursinus*) in response to management interventions in a human-changed landscape. *Anim. Conserv.* **20,** 101–109 (2017).

76. Felton, A., Felton, A. M., Wallace, R. B. & Gómez, H. Identification, behavioral observations, and notes on the distribution of the titi monkeys *Callicebus modestus* and *Callicebus olallae*. *Primate Conserv.* **20,** 41–46 (2006).

77. Ferrari, S. F. *et al.* in *Primates in fragments* (Marsh, L. K. ed.) 123–144 (Springer, 2003).

78. Fimbel, C. Ecological correlates of species success in modified habitats may be disturbance-and site-specific: the primates of Tiwai Island. *Conserv. Biol.* **8,** 106–113 (1994).

79. Fimbel, C. The relative use of abandoned farm clearings and old forest habitats by primates and a forest antelope at Tiwai, Sierra Leone, West Africa. *Biol. Conserv.* **70,** 277–286 (1994).

80. Flesher, K. M. The distribution, habitat use, and conservation status of three Atlantic forest monkeys (*Sapajus xanthosternos*, *Callicebus melanochir*, *Callithrix* sp.) in an agroforestry/forest mosaic in Southern Bahia, Brazil. *Int. J. Primatol.* **36,** 1172–1197 (2015).

81. Freitas, C. H. D., Setz, E. Z., Araújo, A. R. & Gobbi, N. Agricultural crops in the diet of bearded capuchin monkeys, *Cebus libidinosus* Spix (Primates: Cebidae), in forest fragments in southeast Brazil. *Rev. Bras. Zool.* **25,** 32–39 (2008).

82. Fuentes, A., Shaw, E. & Cortes, J. Qualitative assessment of macaque tourist sites in Padangtegal, Bali, Indonesia, and the upper rock nature reserve, Gibraltar. *Int. J. Primatol.* **28,** 1143–1158 (2007).

83. Fungo, B., Eilu, G., Tweheyo, M. & Baranga, D. Forest disturbance and cropping mixtures influence crop raiding by red-tailed monkey and grey-cheeked mangabey around Mabira Forest Reserve, Uganda. *J. Ecol. Nat. Environ.* **5,** 14–23 (2013).

84. Galat-Luong, A. in *Pirang. Ecological investigations in a forest island in the Gambia. Hambourg: stiftung walderhatung in Africa and Bunderforschunsanstalt für forst und holzwirtschaft* (Ellenberg, H. et al. eds.) 187–207 (Warnke Verlag, 1988).

85. Gandhi, A. Catch me if you can: monkey capture in Delhi. *Ethnography* **13,** 43–56 (2012).

86. Ganzhorn, J. U. & Schmid, J. Different population dynamics of *Microcebus murinus* in primary and secondary deciduous dry forests of Madagascar. *Int. J. Primatol.* **19,** 785–796 (1998).

87. Ganzhorn, J. U. A possible role of plantations for primate conservation in Madagascar. *Am. J. Primatol.* **12,** 205–215 (1987).

88. Garcia, J. E. & Mba, J. Distribution, status and conservation of primates in Monte Alen National Park, equatorial Guinea. *Oryx* **31,** 67–76 (1997).

89. Gibrail, M. M. *et al.* Detection of antibodies to Oropouche virus in non-human primates in Goiânia City, Goiás. *Rev. Soc. Bras. Med. Tro.* **49,** 357–360 (2016).

90. Gilbert, K. A. in *Primates in fragments* (Marsh, L. K. ed.) 145–157 (Springer, 2003).

91. Gilhooly, L. J., Rayadin, Y. & Cheyne, S. M. A comparison of hylobatid survey methods using triangulation on Müller's gibbon (*Hylobates muelleri*) in Sungai Wain Protection Forest, East Kalimantan, Indonesia. *Int. J. Primatol.* **36,** 567–582 (2015).

92. Gippoliti, S. & Dell'Omo, G. Primates of the Cantanhez forest and the Cacine basin, Guinea-Bissau. *Oryx* **30,** 74–80 (1996).

93. Goldberg, T. L., Gillespie, T. R. & Rwego, I. B. in *Science and conservation in African forests: how long-term research promotes habitat protection* (Wrangham, R. & Ross, E. eds.) 75-87 (Cambridge University Press, 2008).

94. Goldberg, T. L., Gillespie, T. R., Rwego, I. B., Estoff, E. L. & Chapman, C. A. Forest fragmentation as cause of bacterial transmission among nonhuman primates, humans, and livestock, Uganda. *Emerg. Infect. Dis.* **14,** 1375 (2008).

95. Gómez-Marin, F. *et al.* Food resources and the survival of a group of howler monkeys (*Alouatta palliata mexicana*) in disturbed and restricted habitat at Los Tuxtlas, Veracruz, Mexico. *Neotrop. Primates* **9,** 60 (2001).

96. Gonzalez-Kirchner, J. P. & de la Maza, M. S. Primates hunting by *Guaymi amerindians* in Costa Rica. *Human Evolution* **13,** 15–19 (1998).

97. Goulart, V. D., Teixeira, C. P. & Young, R. J. Analysis of callouts made in relation to wild urban marmosets (*Callithrix penicillata*) and their implications for urban species management. *Eur. J. Wildlife Res.* **56,** 641–649 (2010).

98. Green, K. M. Primate censusing in northern Colombia: a comparison of two techniques. *Primates* **19,** 537–550 (1978).

99. Gupta, A. K. & Kumar, A. Feeding ecology and conservation of the Phayre's leaf monkey *Presbytis phayrei* in northeast India. *Biol. Conserv.* **69,** 301–306 (1994).

100. Guzmán, A., Link, A., Castillo, J. A. & Botero, J. E. Agroecosystems and primate

conservation: shade coffee as potential habitat for the conservation of Andean night monkeys in the northern Andes. *Agric. Ecosyst. Environ.* **215,** 57–67 (2016).

101. Halloran, A. R., Cloutier, C. T., Monde, S. & Sesay, P. B. The tonkolili chimpanzee project in Sierra Leone: implications for chimpanzee conservation strategies in anthropogenic landscapes. *Afr. Primates* **9,** 15–22 (2014).

102. Hasan, M. K. *et al.* Distribution of rhesus macaques (*Macaca mulatta*) in Bangladesh: inter-population variation in group size and composition. *Primate Conserv.* **26,** 125–132 (2013).

103. Heiduck, S. The use of disturbed and undisturbed forest by masked titi monkeys *Callicebus personatus melanochir* is proportional to food availability. *Oryx* **36,** 133- (2002).

104. Henzi, S. P., Brown, L. R., Barrett, L. & Marais, A. J. Troop size, habitat use, and diet of chacma baboons (*Papio hamadryas ursinus*) in commercial pine plantations: implications for management. *Int. J. Primatol.* **32,** 1020–1032 (2011).

105. Hill, C. M. Conflict of interest between people and baboons: crop raiding in Uganda. *Int. J. Primatol.* **21,** 299–315 (2000).

106. Hill, C. M. Crop-raiding by wild vertebrates: the farmer's perspective in an agricultural community in western Uganda. *Int. J. Pest Manage.* **43,** 77–84 (1997).

107. Hockings, K. J. & Sousa, C. Differential utilization of cashew—a low-conflict crop—by sympatric humans and chimpanzees. *Oryx* **46,** 375–381 (2012).

108. Hoffman, T. S. & O'Riain, M. J. Landscape requirements of a primate population in a human-dominated environment. *Front. Zool.* **9,** 1 (2012).

109. Hoffman, T. S. & O'Riain, M. J. Monkey management: using spatial ecology to understand the extent and severity of human–baboon conflict in the Cape Peninsula, South Africa. *Ecol. Soc.* **17,** 13 (2012).

110. Hoffman, T. S. & O'Riain, M. J. The spatial ecology of chacma baboons (*Papio ursinus*) in a human-modified environment. *Int. J. Primatol.* **32,** 308–328 (2011).

111. Hsu, M. J., Kao, C. C. & Agoramoorthy, G. Interactions between visitors and Formosan macaques (*Macaca cyclopis*) at Shou-Shan Nature Park, Taiwan. *Am. J. Primatol.* **71,** 214–222 (2009).

112. Imaki, H., Koganezawa, M. & Maruyama, N. Habitat selection and forest edge use by Japanese monkeys in the Nikko and Imaichi area, central Honshu, Japan. *Biosph. Conserv. Nature, Wildlife, Humans* **7,** 87–96 (2006).

113. Iwaki, T., Okada, T., Seki, K., Izawa, K. & Sakurai, F. *Ogmocotyle ailuri* (Price, 1954)(Digenea: Notocotylidae) found in the Japanese monkey, *Macaca fuscata*. *J. Vet. Med. Sci.* **74,** 1211–1212 (2012).

114. Jaman, M. F. & Huffman, M. A. The effect of urban and rural habitats and resource type on activity budgets of commensal rhesus macaques (*Macaca mulatta*) in

Bangladesh. *Primates* **54,** 49–59 (2013).

115. Johns, A. G. & Johns, B. G. Tropical forest primates and logging: long-term coexistence? *Oryx* **29,** 205–211 (1995).

116. Jung, L., Mourthe, I., Grelle, C. E., Strier, K. B. & Boubli, J. P. Effects of local habitat variation on the behavioral ecology of two sympatric groups of brown howler monkey (*Alouatta clamitans*). *PLoS One* **10,** e0129789 (2015).

117. Kaplan, B. S., O'Riain, M. J., van Eeden, R. & King, A. J. A low-cost manipulation of food resources reduces spatial overlap between baboons (*Papio ursinus*) and humans in conflict. *Int. J. Primatol.* **32,** 1397–1412 (2011).

118. Kaplin, B. A. Ranging behavior of two species of guenons (*Cercopithecus lhoesti* and *C. mitis doggetti*) in the Nyungwe Forest Reserve, Rwanda. *Int. J. Primatol.* **22,** 521–548 (2001).

119. Karanth, K. U. & Sunquist, M. E. Population structure, density and biomass of large herbivores in the tropical forests of Nagarahole, India. *J. Trop. Ecol.* **8,** 21–35 (1992).

120. Kavanagh, M. Invasion of the forest by an African savannah monkey: behavioural adaptations. *Behaviour* **73,** 238–260 (1980).

121. Kifle, Z., Belay, G., & Bekele, A. Population size, group composition and behavioral ecology of geladas (*Theropithecus gelada*) and human-gelada conflict in Wonchit Valley, Ethiopia. *Pak. J. Biol. Sci.* **16,** 1248–1259 (2013).

122. Kool, K. M. The diet and feeding behavior of the silver leaf monkey (*Trachypithecus auratus sondaicus*) in Indonesia. *Int. J. Primatol.* **14,** 667–700 (1993).

123. Kulp, J. & Heymann, E. W. Ranging, activity budget, and diet composition of red titi monkeys (*Callicebus cupreus*) in primary forest and forest edge. *Primates* **56,** 273–278 (2015).

124. Kumar, R., Sinha, A. & Radhakrishna, S. Comparative demography of two commensal macaques in India: implications for population status and conservation. *Folia Primatol.* **84,** 384–393 (2013).

125. Kumar, V., Sankhyan, V. & Thakur, A. Ophthalmic diseases and disorders in free-ranging rhesus macaque (*Macaca mulatta*) of Shivalik hill area of Himachal Pradesh, Northern India. *J. Med. Primatol.* **44,** 89–96 (2015).

126. Kupsch, D., Waltert, M. & Heymann, E. W. Forest type affects prey foraging of saddleback tamarins, *Saguinus nigrifrons*. *Primates* **55,** 403–413 (2014).

127. Lacayo, G. Assessing silvery-brown tamarin (*Saguinus leucopus*) presence in four privately owned cattle ranches in Caldas, Colombia. *Canopy J. Primate Conserv.* **16,** 25–27 (2015).

128. LaFleur, M. & Gould, L. Feeding outside the forest: the importance of crop raiding and an invasive weed in the diet of gallery forest ring-tailed lemurs (*Lemur catta*) following a cyclone at the Beza Mahafaly Special Reserve, Madagascar. *Folia Primatol.* **80,** 233–246 (2009).

129. Lazo, F. J. J. M., Culot, L., Huynen, M. C. & Heymann, E. W. Effect of resting patterns of tamarins (*Saguinus fuscicollis* and *Saguinus mystax*) on the spatial distribution of seeds and seedling recruitment. *Int. J. Primatol.* **32,** 223–237 (2011).

130. Lees, A. C. & Peres, C. A. Conservation value of remnant riparian forest corridors of varying quality for Amazonian birds and mammals. *Conserv. Biol.* **22,** 439–449 (2008).

131. Lemessa, D., Hylander, K. & Hambäck, P. Composition of crops and land-use types in relation to crop raiding pattern at different distances from forests. *Agric. Ecosyst. Environ.* **167,** 71–78 (2013).

132. Lemos de Sá, R. M. Situacao de uma populacao de mono-carvoeiro, *Brachyteles arachnoides*, em fragmento de Mata Atlantica (MG), implicacoes para sua conservacao. (1988).

133. Lenz, B. B., Jack, K. M. & Spironello, W. R. Edge effects in the primate community of the biological dynamics of forest fragments project, Amazonas, Brazil. *Am. J. Phys. Anthropol.* **155,** 436–446 (2014).

134. Li, D. *et al.* Ranging of *Rhinopithecus bieti* in the Samage Forest, China. II. Use of land cover types and altitudes. *Int. J. Primatol.* **29,** 1147 (2008).

135. Li, Y. The effect of forest clear-cutting on habitat use in Sichuan snub-nosed monkey (*Rhinopithecus roxellana*) in Shennongjia Nature Reserve, China. *Primates* **45,** 69–72 (2004).

136. Liebsch, D. & Mikich, S. B. First record of *Eucalyptus spp.* bark-stripping by Brown-capuchin monkeys (*Sapajus nigritus*, Primates: Cebidae). *Ciência Florest.* **25,** 501–505 (2015).

137. Lindshield, S. M. in *Ethnoprimatology* (Dore, K. M., Riley, E. P. & A. Fuentes eds.) 351–369 (Cambridge University Press, 2016).

138. Lokschin, L. X., Rodrigo, C. P., Hallal Cabral, J. N. & Buss, G. Power lines and howler monkey conservation in Porto Alegre, Rio Grande do Sul, Brazil. *Neotrop. Primates* **14,** 76–80 (2007).

139. Luckett, J., Danforth, E., Linsenbardt, K. & Pruetz, J. Planted trees as corridors for primates at El Zota Biological Field Station, Costa Rica. *Neotrop. Primates* **12,** 143–146 (2004).

140. Lwanga, J. S. Spatial distribution of primates in a mosaic of colonizing and old growth forest at Ngogo, Kibale National Park, Uganda. *Primates* **47,** 230–238 (2006).

141. Maibeche, Y., Moali, A., Yahi, N. & Menard, N. Is diet flexibility an adaptive life trait for relictual and peri-urban populations of the endangered primate *Macaca sylvanus*? *PLoS One* **10,** e0118596 (2015).

142. Mallott, E. K. Influences of natural and anthropogenic landscape features on ranging patterns of white-faced capuchins (*Cebus capucinus*). *Am. J. Phys. Anthropol.* **147,**

201–202 (2012).

143. Marchal, V. & Hill, C. Primate crop-raiding: a study of local perceptions in four villages in North Sumatra, Indonesia. *Primate Conserv.* **24,** 107–116 (2009).

144. Martinez, B. T. & Razafindratsima, O. H. Frugivory and seed dispersal patterns of the red-ruffed lemur, *Varecia rubra,* at a forest restoration site in Masoala National Park, Madagascar. *Folia Primatol.* **85,** 228–243 (2014).

145. Mass, V. *et al.* Lemur bridges provide crossing structures over roads within a forested mining concession near Moramanga, Toamasina Province, Madagascar. *Conserv. Evid.* **8,** 11–18 (2011).

146. Massussi, J. A., Djieto-Lordon, C., Njiokou, F., Laveissière, C. & van der Ploeg, J. D. Influence of habitat and seasonal variation on wild mammal diversity and distribution with special reference to the *Trypanosoma brucei* gambiense host-reservoir in Bipindi (Cameroon). *Acta Trop.* **112,** 308–315 (2009).

147. Mathur, R. & Manohar, B. R. Density of *Macaca mulatta* and *Presbytis entellus* in the old city of Jaipur: a three year survey. *Appl. Anim. Behav. Sci.* **27,** 351–361 (1990).

148. Matthews, A. & Matthews, A. Survey of gorillas (*Gorilla gorilla gorilla*) and chimpanzees (*Pan troglodytes troglodytes*) in Southwestern Cameroon. *Primates* **45,** 15–24 (2004).

149. McCann, C. *et al.* in *Primates in fragments* (Marsh, L. K. ed.) 321–341 (Springer, 2003).

150. McKinney, T. A classification system for describing anthropogenic influence on nonhuman primate populations. *Am. J. Primatol.* **77,** 715–726 (2015).

151. McKinney, T. Anthropogenic change and primate predation risk: crested caracaras (*Caracara plancus*) attempt predation on mantled howler monkeys (*Alouatta palliata*). *Neotrop. Primates* **16,** 24–27 (2009).

152. McKinney, T. The effects of provisioning and crop-raiding on the diet and foraging activities of human-commensal white-faced capuchins (*Cebus capucinus*). *Am. J. Primatol.* **73,** 439–448 (2011).

153. McLennan, M. R. & Plumptre, A. J. Protected apes, unprotected forest: composition, structure and diversity of riverine forest fragments and their conservation value in Uganda. *Trop. Conserv. Sci.* **5,** 79–103 (2012).

154. McPherson, S. C., Brown, M. & Downs, C. T. Diet of the crowned eagle (*Stephanoaetus coronatus*) in an urban landscape: potential for human-wildlife conflict? *Urban Ecosyst.* **19,** 383–396 (2016).

155. Md-Zain, B. M. *et al.* A comprehensive population survey and daily activity budget on long-tailed macaques of Universiti Kebangsaan Malaysia. *J. Biol. Sci.* **10,** 608–615 (2010).

156. Mekonnen, A. *et al.* Newly discovered bale monkey populations in forest fragments in

southern Ethiopia: evidence of crop raiding, hybridization with grivets, and other conservation threats. *Am. J. Primatol.* **74,** 423–432 (2012).

157. Méndez-Carvajal, P. G. Population size, distribution and conservation status of howler monkeys (*Alouatta coibensis trabeata*) and spider monkeys (*Ateles geoffroyi azuerensis*) on the Azuero Peninsula, Panama. *Primate Conserv.* **26,** 3–15 (2013).

158. Merker, S. & Yustian, I. Habitat use analysis of Dian's tarsier (*Tarsius dianae*) in a mixed-species plantation in Sulawesi, Indonesia. *Primates* **49,** 161–164 (2008).

159. Merker, S., Yustian, I. & Mühlenberg, M. Responding to forest degradation: altered habitat use by Dian's tarsier *Tarsius dianae* in Sulawesi, Indonesia. *Oryx* **39,** 189–195 (2005).

160. Mikich, S. B. & Liebsch, D. Assessment of food supplementation and surveillance as techniques to reduce damage caused by black capuchin monkeys *Sapajus nigritus* to forest plantations. *Curr. Zool.* **60,** 581–590 (2014).

161. Mikich, S. B. & Liebsch, D. Damage to forest plantations by tufted capuchins (*Sapajus nigritus*): too many monkeys or not enough fruits? *Forest Ecol. Manage.* **314,** 9–16 (2014).

162. Mohnot, S. M., Gadgil, M. & Makwana, S. C. On the dynamics of the hanuman langur populations of Jodhpur (Rajasthan, India). *Primates* **22,** 182–191 (1981).

163. Moore, R. S., Nekaris, K. A. I. & Eschmann, C. Habitat use by western purple-faced langurs *Trachypithecus vetulus nestor* (Colobinae) in a fragmented suburban landscape. *Endanger. Species Res.* **12,** 227–234 (2010).

164. Mulu, K. S. *Are the endemic and endangered Tana River primates culprits of crop raiding? Evaluating human–nonhuman primate conflict status around Tana river primate reserve, in Kenya*. 34 (2010).

165. Muñoz, D., Estrada, A., Naranjo, E. & Ochoa, S. Foraging ecology of howler monkeys in a cacao (*Theobroma cacao*) plantation in Comalcalco, Mexico. *Am. J. Primatol.* **68,** 127–142 (2006).

166. Narvaez-Rivera, G. Bridging the forest gap: an experimental evaluation of bridges for new world monkeys. in *Symposium on undergraduate research and creative expression* (2014).

167. Nasi, R., Koponen, P., Poulsen, J. G., Buitenzorgy, M. & Rusmantoro, W. Impact of landscape and corridor design on primates in a large-scale industrial tropical plantation landscape. *Biodivers. Conserv.* **17,** 1105–1126 (2008).

168. Naughton-Treves, L. Predicting patterns of crop damage by wildlife around Kibale National Park, Uganda. *Conserv. Biol.* **12,** 156–168 (1998).

169. Neville, M. K. The population structure of red howler monkeys (*Alouatta seniculus*) in Trinidad and Venezuela. *Folia Primatol.* **17,** 56–86 (1972).

170. Nijman, V. Effects of habitat disturbance and hunting on the density and the biomass

of the endemic Hose's leaf monkey *Presbytis hosei* (Thomas, 1889) (Mammalia: Primates: Cercopithecidae) in east Borneo. *Contrib. Zool.* **73,** 283–291 (2004).

171. Nijman, V. Geographic distribution of ebony leaf monkey *Trachypithecus auratus*. *Contrib. Zool.* **69,** 157–177 (2000).

172. Norscia, I. & Palagi, E. Berenty 2006: census of *Propithecus verreauxi* and possible evidence of population stress. *Int. J. Primatol.* **29,** 1099 (2008).

173. Nowak, K. & Lee, P. C. in *Primates in fragments. Complexity and resilience* (Marsh, L. K. & Chapman, C. A. eds.) 199–211 (Springer, 2013).

174. Oates, J. F. The diet of the olive colobus monkey, *Procolobus verus*, in Sierra Leone. *Int. J. Primatol.* **9,** 457–478 (1988).

175. Oberste, M. S. *et al.* Characterizing the picornavirus landscape among synanthropic nonhuman primates in Bangladesh, 2007–2008. *J. Virol.* **92,** 1–44 (2012).

176. Oliveira, L. C. & Dietz, J. M. Predation risk and the interspecific association of two Brazilian Atlantic forest primates in Cabruca agroforest. *Am. J. Primatol.* **73,** 852–860 (2011).

177. Oliveira, L. C., Neves, L. G., Raboy, B. E. & Dietz, J. M. Abundance of jackfruit (*Artocarpus heterophyllus*) affects group characteristics and use of space by golden-headed lion tamarins (*Leontopithecus chrysomelas*) in cabruca agroforest. *Environ. Manage.* **48,** 248–262 (2011).

178. Omar, A. & De Vos, A. Damage to exotic softwoods by Sykes monkeys (*Cercopithecus mitis* Kolbi Neuman). *E. Afr. Agr. Forestry J.* **35,** 323–330 (1970).

179. Onderdonk, D. A. & Chapman, C. A. Coping with forest fragmentation: the primates of Kibale National Park, Uganda. *Int. J. Primatol.* **21,** 587–611 (2000).

180. Oppenheimer, J. R. *Presbytis entellus*: birth in a free-ranging primate troop. *Primates* **17,** 541–542 (1976).

181. Papworth, S. & Mejia, M. Population density of Ecuadorian mantled howler monkeys (*Alouatta palliata aequatorialis*) in a tropical dry forest, with information on habitat selection, calling behavior and cluster sizes. *Stud. Neotrop. Fauna E.* **50,** 65–72 (2015).

182. Parker, L., Nijman, V. & Nekaris, K. A. I. When there is no forest left: fragmentation, local extinction, and small population sizes in the Sri Lankan western purple-faced langur. *Endanger. Species Res.* **5,** 29–36 (2008).

183. Parry, L., Barlow, J. & Peres, C. A. Large-vertebrate assemblages of primary and secondary forests in the Brazilian Amazon. *J. Trop. Ecol.* **23,** 653–662 (2007).

184. Parry, L., Barlow, J. O. S. & Peres, C. A. Hunting for sustainability in tropical secondary forests. *Conserv. Biol.* **23,** 1270–1280 (2009).

185. Patterson, L., Kalle, R. & Downs, C. Predation of artificial bird nests in suburban gardens of KwaZulu-Natal, South Africa. *Urban Ecosyst.* **19,** 615–630 (2016).

186. Pazol, K. & Cords, M. Seasonal variation in feeding behavior, competition and female social relationships in a forest dwelling guenon, the blue monkey (*Cercopithecus mitis stuhlmanni*), in the Kakamega Forest, Kenya. *Behav. Ecol. Sociobiol.* **58,** 566–577 (2005).

187. Pebsworth, P. A., MacIntosh, A. J., Morgan, H. R. & Huffman, M. A. Factors influencing the ranging behavior of chacma baboons (*Papio hamadryas ursinus*) living in a human-modified habitat. *Int. J. Primatol.* **33,** 872–887 (2012).

188. Pebsworth, P. A., Morgan, H. R. & Huffman, M. A. Evaluating home range techniques: use of Global Positioning System (GPS) collar data from chacma baboons. *Primates* **53,** 345–355 (2012).

189. Peng-Fei, F., Xue-Long, J. & Chang-Cheng, T. The critically endangered black crested gibbon *Nomascus concolor* on Wuliang Mountain, Yunnan, China: the role of forest types in the species' conservation. *Oryx* **43,** 203–208 (2009).

190. Pérez, L. & Pacheco, L. F. Wildlife damage mitigation in agricultural crops in a Bolivian montane forest. *Rev. Biol. Trop.* **62,** 1495–1507 (2014).

191. Perez-Elissetche, G. K. Suplementación dietética de monos araña (*Ateles geoffroyi*) en paisajes fragmentados (2015).

192. Pienkowski, M. W. *et al.* Temporal patterns of crop-raiding by primates: linking food availability in croplands and adjacent forest. *J. Appl. Ecol.* **35,** 596–606 (1998).

193. Pinto, L. P. S., Costa, C. M., Strier, K. B. & da Fonseca, G. A. Habitat, density and group size of primates in a Brazilian tropical forest. *Folia Primatol.* **61,** 135–143 (1993).

194. Pinzón, X. C. in *Primatología en Colombia: avances al principo del milenio* (Pereira-Bengoa, V., Stevenson, P. R., Bueno, M. L. & Nassar-Montoya, F. eds.) 91–97 (Fundación Universitaria San Martín, 2010).

195. Pirta, R. S. Maintenance of cooperative life in forest and urban rhesus monkeys (*Macaca mulatta*). *Proc. Anim. Sci.* **93,** 83–89 (1984).

196. Pozo-Montuy, G. & Serio-Silva, J. C. Movement and resource use by a group of *Alouatta pigra* in a forest fragment in Balancán, México. *Primates* **48,** 102–107 (2007).

197. Pozo-Montuy, G., Serio-Silva, J. C. & Bonilla-Sánchez, Y. M. Influence of the landscape matrix on the abundance of arboreal primates in fragmented landscapes. *Primates* **52,** 139–147 (2011).

198. Pozo-Montuy, G., Serio-Silva, J. C., Chapman, C. A. & Bonilla-Sánchez, Y. M. Resource use in a landscape matrix by an arboreal primate: evidence of supplementation in black howlers (*Alouatta pigra*). *Int. J. Primatol.* **34,** 714–731 (2013).

199. Priston, N. E. Exclosure plots as a mechanism for quantifying damage to crops by primates. *Int. J. Pest Manage.* **55,** 243–249 (2009).

200. Priston, N. E., Wyper, R. M. & Lee, P. C. Buton macaques (*Macaca ochreata brunnescens*): crops, conflict, and behavior on farms. *Am. J. Primatol.* **74,** 29–36 (2012).

201. Raboy, B. E., Christman, M. C. & Dietz, J. M. The use of degraded and shade cocoa forests by endangered golden-headed lion tamarins *Leontopithecus chrysomelas*. *Oryx* **38,** 75–83 (2004).

202. Ramakrishnan, U. & Coss, R. G. Recognition of heterospecific alarm vocalization by bonnet macaques (*Macaca radiata*). *J. Comp. Psychol.* **114,** 3 (2000).

203. Ramos-Fernández, G. & Ayala-Orozco, B. in *Primates in fragments: ecology and conservation* (Marsh, L. K. ed.) 191–209 (Springer, 2003).

204. Ramos-Fernández, G. *et al.* Lévy walk patterns in the foraging movements of spider monkeys (*Ateles geoffroyi*). *Behav. Ecol. Sociobiol.* **55,** 223–230 (2004).

205. Ramos-Fernández, G., Vick, L. G., Aureli, F., Schaffner, C. & Taub, D. M. Behavioral ecology and conservation status of spider monkeys in the Otoch Ma'ax Yetel Kooh protected area. *Neotrop. Primates* **11,** 157–160 (2003).

206. Rayadin, Y. & Spehar, S. N. Body mass of wild bornean orangutans living in human-dominated landscapes: implications for understanding their ecology and conservation. *Am. J. Phys. Anthropol.* **157,** 339–346 (2015).

207. Regmi, G. R., Nekaris, K. A. I., Kandel, K. & Nijman, V. Crop-raiding macaques: predictions, patterns and perceptions from Langtang National Park, Nepal. *Endanger. Species Res.* **20,** 217–226 (2013).

208. Riley, E. P. & Priston, N. E. Macaques in farms and folklore: exploring the human-nonhuman primate interface in Sulawesi, Indonesia. *Am. J. Primatol.* **72,** 848–854 (2010).

209. Rocancio, N., Rojas, W. & Estévez-Vinasco., J. Densidad poblacional y tamaño de grupo de *Saguinus leucopus* en parches de bosque en el departamento de Caldas, Colombia. *Neotrop. Primates* **15,** 63–67 (2008).

210. Rodrigues, N. N. & Martinez, R. A. Wildlife in our backyard: interactions between wied's marmoset *Callithrix kuhlii* (Primates: Callithrichidae) and residents of Ilheus, Bahia, Brazil. *Wildlife Biol.* **20,** 91–96 (2014).

211. Roscoe, C. J., de Silva, M. A., Hapuarachchi, N. C. & Krishantha, P. R. A new color morph of the southern purple-faced langur (*Semnopithecus vetulus vetulus*) from the rainforests of Southwestern Sri Lanka. *Primate Conserv.* **26,** 115–124 (2013).

212. Rosembaum, B. Population densities of Sulawesi crested black macaques on Bacan and Sulawesi: effects of habitat disturbances and hunting. *Am. J. Primatololgy* **44,** 89–106 (1998).

213. Ross, C., Srivastava, A. & Pirta, R. S. Human influences on the population density of Hanuman langurs *Presbytis entellus* and rhesus macaques *Macaca mulatta* in Shimla,

India. *Biol. Conserv.* **65,** 159–163 (1993).

214. Rowell, T. E. Forest living baboons in Uganda. *J. Zool.* **149,** 344–364 (1966).

215. Rudran, R., Dayananda, H. G. S. K., Jayamanne, D. D. & Sirimanne, D. G. R. Food habits and habitat use patterns of Sri Lanka's western purple-faced langur. *Primate Conserv.* **27,** 99–108 (2013).

216. Russon, A. E., Kuncoro, P. & Ferisa, A. Orangutan behavior in Kutai National Park after drought and fire damage: adjustments to short-and long-term natural forest regeneration. *Am. J. Primatol.* **77,** 1276–1289 (2015).

217. Ryan, S. J. & Hartter, J. Beyond ecological success of corridors: integrating land use history and demographic change to provide a whole landscape perspective. *Ecol. Restor.* **30,** 320–328 (2012).

218. Ryan, S. J. *et al.* A survey of gastrointestinal parasites of olive baboons (*Papio anubis*) in human settlement areas of Mole National Park, Ghana. *J. Parasitol.* **98,** 885–888 (2012).

219. Saikia, N. Status of sympatric langurs in a tea plantation in Barak valley, Assam, India. *Conserv. Rev.* **5,** 1 (2014).

220. Saj, T. L., Sicotte, P. & Paterson, J. D. The conflict between vervet monkeys and farmers at the forest edge in Entebbe, Uganda. *Afr. J. Ecol.* **39,** 195–199 (2001).

221. Sakamaki, H., Enari, H., Aoi, T. & Kunisaki, T. Winter food abundance for Japanese monkeys in differently aged Japanese cedar plantations in snowy regions. *Mamm. Study* **36,** 1–10 (2011).

222. Scheun, J., Bennett, N. C., Ganswindt, A. & Nowack, J. The hustle and bustle of city life: monitoring the effects of urbanisation in the African lesser bushbaby. *Sci. Nat.* **102,** 57 (2015).

223. Schleuning, M. *et al.* Specialization and interaction strength in a tropical plant-frugivore network differ among forest strata. *Ecology* **92,** 26–36 (2011).

224. Schwitzer, N. *et al.* Parasite prevalence in blue-eyed black lemurs *Eulemur flavifrons* in differently degraded forest fragments. *Endanger. Species Res.* **12,** 215–225 (2010).

225. Sha, J. C. M. *et al.* Status of the long-tailed macaque *Macaca fascicularis* in Singapore and implications for management. *Biodivers. Conserv.* **18,** 2909–2926 (2009).

226. Singh, M. & Rao, N. R. Population dynamics and conservation of commensal bonnet macaques. *Int. J. Primatol.* **25,** 847–859 (2004).

227. Singh, M. & Vinathe, S. Inter-population differences in the time budgets of bonnet monkeys (*Macaca radiata*). *Primates,* **31,** 589–596 (1990).

228. Singh, M., Erinjery, J. J., Kavana, T. S., Roy, K. & Singh, M. Drastic population decline and conservation prospects of roadside dark-bellied bonnet macaques (*Macaca radiata radiata*) of southern India. *Primates* **52,** 149–154 (2011).

229. Slocum, M. G. & Horvitz, C. C. Seed arrival under different genera of trees in a neotropical pasture. *Plant Ecol.* **149,** 51–62 (2000).

230. Sorensen, T. C. & Fedigan, L. M. Distribution of three monkey species along a gradient of regenerating tropical dry forest. *Biol. Conserv.* **92,** 227–240 (2000).

231. Southwick, C. H. & Siddiqi, M. F. Primate commensalism: the rhesus monkey in India. in *XIVe congrès de la Société internationale de Primatologie* (Société nationale de protection de la nature et d'acclimatation de France, Paris (FRA), 1994).

232. Southwick, C. H., Beg, M. A. & Siddiqi, M. R. A population survey of rhesus monkeys in villages, towns and temples of northern India. *Ecology* **42,** 538–547 (1961).

233. Southwick, C. H., Siddioi, M. F., Farooqui, M. Y. & Pal, B. C. Effects of artificial feeding on aggressive behaviour of rhesus monkeys in India. *Anim. Behav.* **24,** 11–15 (1976).

234. Stoner, K. E. Prevalence and intensity of intestinal parasites in mantled howling monkeys (*Alouatta palliata*) in Northeastern Costa Rica: implications for conservation biology. *Conserv. Biol.* **10,** 539–546 (1996).

235. Struhsaker, T. T. Ecology of vervet monkeys (*Cercopithecus aethiops*) in The Masai-Amboseli Game Reserve, Kenya. *Ecology* **48,** 891–904 (1967).

236. Teichroeb, J. A., Kutz, S. J., Parkar, U., Thompson, R. C. & Sicotte, P. Ecology of the gastrointestinal parasites of *Colobus vellerosus* at Boabeng-Fiema, Ghana: possible anthropozoonotic transmission. *Am. J. Phys. Anthropol.* **140,** 498–507 (2009).

237. Teixeira, B. *et al.* Good neighbours: distribution of black-tufted marmoset (*Callithrix penicillata*) in an urban environment. *Wildl. Res.* **42,** 579–589 (2015).

238. Teixeira, F. Z., Printes, R. C., Fagundes, J. C. G., Alonso, A. C. & Kindel, A. Canopy bridges as road overpasses for wildlife in urban fragmented landscapes. *Biota Neotrop.* **13,** 117–123 (2013).

239. Thomas, S. C. Population densities and patterns of habitat use among anthropoid primates of the Ituri Forest, Zaire. *Biotropica* **23,** 68–83 (1991).

240. Tisovec, K. C., Cassano, C. R., Boubli, J. P. & Pardini, R. Mixed-species groups of marmosets and tamarins across a gradient of agroforestry intensification. *Biotropica* **46,** 248–255 (2014).

241. Tweheyo, M., Hill, C. M. & Obua, J. Patterns of crop raiding by primates around the Budongo Forest Reserve, Uganda. *Wildlife Biol.* **11,** 237–247 (2005).

242. Urquiza-Haas, T., Peres, C. A. & Dolman, P. M. Regional scale effects of human density and forest disturbance on large-bodied vertebrates throughout the Yucatán Peninsula, Mexico. *Biol. Conserv.* **142,** 134–148 (2009).

243. Valenca-Silva, G. *et al.* Reporting social behaviours of mixed-species troops formed by *Callithrix jacchus* and *Callithrix penicillata* (Primate, Callitrichidae). *Braz. J. Biol.* **74,** 607–611 (2014).

244. Valladares-Padua, C., Cullen, J. L. & Padua, S. A pole bridge to avoid primate road kills. *Neotrop. Primates* **3,** 13–15 (1995).

245. Voskamp, A., Rode, E. J., Coudrat, C. N., Wilson, R. J. & Nekaris, K. A. I. Modelling the habitat use and distribution of the threatened Javan slow loris *Nycticebus javanicus*. *Endanger. Species Res.* **23,** 277–286 (2014).

246. Vulinec, K., Lambert, J. E. & Mellow, D. J. Primate and dung beetle communities in secondary growth rain forests: implications for conservation of seed dispersal systems. *Int. J. Primatol.* **27,** 855–879 (2006).

247. Waite, T. A., Chhangani, A. K., Campbell, L. G., Rajpurohit, L. S. & Mohnot, S. M. Sanctuary in the city: urban monkeys buffered against catastrophic die-off during ENSO-related drought. *Ecohealth* **4,** 278–286 (2007).

248. Wallace, G. E. & Hill, C. M. Crop damage by primates: quantifying the key parameters of crop-raiding events. *PLoS One* **7,** e46636, doi: 10.1371/journal.pone.0046636 (2012).

249. Wenz, A., Heymann, E. W., Petney, T. N. & Taraschewski, H. F. The influence of human settlements on the parasite community in two species of Peruvian tamarin. *Parasitology* **137,** 675–684 (2010).

250. Wiafe, E. D. & Arku, F. S. Victims' perspectives of lowe's monkeys' (*Cercopithecus campbelli lowei*) crop raiding events in Ghana: a case of Buabeng-Fiema Monkey Sanctuary. *J. Biodivers. Environ. Sci.* **2,** 1–8 (2012).

251. Wieczkowski, J. & Kinnaird, M. Shifting forest composition and primate diets: a 13-year comparison of the Tana River mangabey and its habitat. *Am. J. Primatol.* **70,** 339–348 (2008).

252. Williams, H. E. & Vaughan, C. White-faced monkey (*Cebus capucinus*) ecology and management in Neotropical agricultural landscapes during the dry season. *Rev. Biol. Trop.* **49,** 1199–1206 (2001).

253. Williams-Guillén, K., McCann, C., Martínez Sánchez, J. C. & Koontz, F. Resource availability and habitat use by mantled howling monkeys in a Nicaraguan coffee plantation: can agroforests serve as core habitat for a forest mammal? *Anim. Conserv.* **9,** 331–338 (2006).

254. Williams-Guillén, K., Otterstrom, S. M. & Gomez, C. *Assessment of fragmented landscapes as habitat for the Central American spider monkey (*Ateles geoffroyi*) in southwestern Nicaragua*. 18 (2008).

255. Xiang, Z. F., Huo, S. & Xiao, W. Habitat selection of black-and-white snub-nosed monkeys (*Rhinopithecus bieti*) in Tibet: implications for species conservation. *Am. J. Primatol.* **73,** 347–355 (2011).

256. Yamada, A. & Muroyama, Y. Effects of vegetation type on habitat use by crop-raiding japanese macaques during a food-scarce season. *Primates* **51,** 159–166 (2010).

257. Yamagiwa, J., Mwanza, N., Yumoto, T. & Maruhashi, T. Ant eating by eastern lowland gorillas. *Primates* **32,** 247–253 (1991).

258. Zárate, D. A., Andresen, E., Estrada, A. & Serio-silva, J. C. Black howler monkey (*Alouatta pigra*) activity, foraging and seed dispersal patterns in shaded cocoa plantations versus rainforest in southern Mexico. *Am. J. Primatol.* **76,** 890–899 (2014).
