# Supplementary material for: The conservation value of human-modified landscapes for the world’s primates
Source: Nat Commun. 2019 Jan 11;10:152. doi: 10.1038/s41467-018-08139-0 (PMC6329842; doi:10.1038/s41467-018-08139-0)
Supplement: Supplementary file 3 — Reporting Summary [file 41467_2018_8139_MOESM3_ESM.pdf]

nature research

Corresponding author(s): Víctor Arroyo-Rodríguez & Carmen Galán-Acedo

Reporting Summary

Nature Research wishes to improve the reproducibility of the work that we publish. This form provides structure for consistency and transparency in reporting. For further information on Nature Research policies, see Authors & Referees and the Editorial Policy Checklist.

Statistical parameters

When statistical analyses are reported, confirm that the following items are present in the relevant location (e.g. figure legend, table legend, main text, or Methods section).

n/a	Confirmed	
☐	☒	The exact sample size (*n*) for each experimental group/condition, given as a discrete number and unit of measurement
☐	☒	An indication of whether measurements were taken from distinct samples or whether the same sample was measured repeatedly
☒	☐	The statistical test(s) used AND whether they are one- or two-sided<br>*Only common tests should be described solely by name; describe more complex techniques in the Methods section.*
☒	☐	A description of all covariates tested
☒	☐	A description of any assumptions or corrections, such as tests of normality and adjustment for multiple comparisons
☒	☐	A full description of the statistics including central tendency (e.g. means) or other basic estimates (e.g. regression coefficient) AND variation (e.g. standard deviation) or associated estimates of uncertainty (e.g. confidence intervals)
☐	☒	For null hypothesis testing, the test statistic (e.g. *F*, *t*, *r*) with confidence intervals, effect sizes, degrees of freedom and *P* value noted<br>*Give P values as exact values whenever suitable.*
☒	☐	For Bayesian analysis, information on the choice of priors and Markov chain Monte Carlo settings
☒	☐	For hierarchical and complex designs, identification of the appropriate level for tests and full reporting of outcomes
☒	☐	Estimates of effect sizes (e.g. Cohen's *d*, Pearson's *r*), indicating how they were calculated
☒	☐	Clearly defined error bars<br>*State explicitly what error bars represent (e.g. SD, SE, CI)*

Our web collection on statistics for biologists may be useful.

Software and code

Policy information about availability of computer code

Data collection	No softaware was used
Data analysis	We used Microsoft Office Excel

For manuscripts utilizing custom algorithms or software that are central to the research but not yet described in published literature, software must be made available to editors/reviewers upon request. We strongly encourage code deposition in a community repository (e.g. GitHub). See the Nature Research guidelines for submitting code & software for further information.

Data

Policy information about availability of data

All manuscripts must include a data availability statement. This statement should provide the following information, where applicable:

- Accession codes, unique identifiers, or web links for publicly available datasets
- A list of figures that have associated raw data
- A description of any restrictions on data availability

All data generated or analysed during this study are included in the article or its Supplementary Information files

Field-specific reporting

Please select the best fit for your research. If you are not sure, read the appropriate sections before making your selection.

☐ Life sciences ☐ Behavioural & social sciences ☒ Ecological, evolutionary & environmental sciences

For a reference copy of the document with all sections, see nature.com/authors/policies/ReportingSummary-flat.pdf

Ecological, evolutionary & environmental sciences study design

All studies must disclose on these points even when the disclosure is negative.

Study description	We provide quantitative evidence regarding which types of anthropic land covers are most frequently used by primates and for what activities. We also evaluate whether there are certain characteristics of the species, such as conservation status, ecological traits, and/or phylogenetic relationships that may help us predict their use of anthropic land covers. We address these questions by reviewing 468 records of anthropic land covers use by primates. We compare the characteristics of species using these anthropic land covers with the expected values based on all of the world's primates.
Research sample	Because for some anthropic land covers the available literature is scarce, we selected the most recent 60 studies per each anthropic land cover type. Nevertheless, as some studies included information about more than one antropic land cover type, the final database included 258 independent studies containing 468 records of 147 primate species using anthropic land covers. Such records span 44 countries from four biogeographic realms: mainland Africa (17 countries), Madagascar, Asia (13 countries), and the Neotropics (13 countries).
Sampling strategy	For each anthropic land cover (i.e. human settlements, open areas, connectors, tree plantations and secondary vegetation) we selected the most recent 60 studies reporting anthropic land cover use by primates. We considered that this sample was large enough to represent the use of anthropic land covers by primates.
Data collection	Data collection was a review of different published studies and grey literature reporting anthropic land covers use by primates. We also recorded ecological traits of all the world primates. C. G.-A. reviewed literature and collected the data.
Timing and spatial scale	Evidences of anthropic land covers use by primates was collected from August 3rd 2016 to November 2nd, 2016. Ecological traits of all the world primates were collected from 10th November 2016 to 15th September 2017.
Data exclusions	We excluded review articles and studies with captive or reintroduced animals
Reproducibility	We did not use an experimental approach
Randomization	We selected the first 60 studies or primates using each of the anthropic land covers categorized (i.e. human settlements, open areas, connectors, tree plantations and secondary vegetation)
Blinding	This study did not require blinding because was a review of studies using key words criteria

Did the study involve field work? ☐ Yes ☒ No

Reporting for specific materials, systems and methods

Materials & experimental systems

n/a	Involved in the study
☒	☐ Unique biological materials
☒	☐ Antibodies
☒	☐ Eukaryotic cell lines
☒	☐ Palaeontology
☒	☐ Animals and other organisms
☒	☐ Human research participants

Methods

n/a	Involved in the study
☒	☐ ChIP-seq
☒	☐ Flow cytometry
☒	☐ MRI-based neuroimaging
